# Integrated Single-Cell and Spatial Transcriptomic Analysis Reveals the Immunoregulatory Role of MIF Signaling in Colorectal Cancer

**DOI:** 10.3390/genes17070817

**Published:** 2026-07-17

**Authors:** Yuxian Liu, Junyuan Zhang, Xiaohui Li, Xingjie Chen, Kangcheng Xu, Yanni Cao

**Affiliations:** 1 School of Artificial Intelligence, Anhui University of Science & Technology, Huainan 232001, China; yxl@aust.edu.cn (Y.L.); 2024201872@aust.edu.cn (J.Z.); 2023201661@aust.edu.cn (X.L.); chenxj340@163.com (X.C.); 2024201843@aust.edu.cn (K.X.); 2 State Key Laboratory of Digital Intelligent Technology for Unmanned Coal Mining, Anhui University of Science & Technology, Huainan 232001, China

**Keywords:** primary colorectal cancer, single-cell RNA sequencing, spatial transcriptomics, tumor microenvironment, immune assessment

## Abstract

**Background**: The cellular heterogeneity and spatial organization patterns of the tumor microenvironment (TME) play a crucial role in colorectal cancer (CRC) progression, but their spatial distribution and cellular communication mechanisms require further elucidation. This study aims to systematically dissect the cellular heterogeneity and spatial organization characteristics of the CRC microenvironment by integrating single-cell and spatial transcriptomic data. **Methods**: Single-cell RNA sequencing and spatial transcriptomics data of primary CRC were integrated to characterize the TME. Intercellular signaling patterns were elucidated through communication analysis, spatial niche clustering, ligand–receptor pair analysis, and spatial expression mapping. The ESTIMATE algorithm was applied to assess the correlation between TME scores and pathway-associated genes. Drug sensitivity prediction was performed using the oncoPredict. **Results**: Single-cell analysis identifies nine major cell types, revealing significant cellular heterogeneity. Intercellular communication analysis demonstrates that the MIF signaling pathway plays a prominent role within the TME communication network, with MIF-(CD74+CD44) and MIF-(CD74+CXCR4) identified as dominant receptor complexes. Spatial transcriptomic analysis reveals distinct spatial functional partitioning of these signaling axes. Signaling flow analysis indicates an immunosuppressive “tumor–immune” axis mediated by MIF signaling from tumor epithelial cells to immune cells. *MIF* expression is negatively correlated with ImmuneScore, while its receptors *CD74*, *CD44*, and *CXCR4* are positively correlated. Drug sensitivity analysis reveals potential associations between key genes (*MIF*, *CD74*, *CD44*, and *CXCR4*) in the MIF pathway and various anti-tumor drugs. **Conclusions**: Our study reveals the cellular heterogeneity of the CRC microenvironment from multiple perspectives, elucidates the key mechanisms of the MIF signaling pathway, and provides potential therapeutic targets for CRC treatment.

## 1. Introduction

Colorectal cancer (CRC) is one of the most common malignancies globally and the second leading cause of cancer-related deaths in the United States [1]. The complexity and heterogeneity of the tumor microenvironment (TME) are key factors influencing disease occurrence, progression, and therapeutic response [2]. TME is composed of various cell types, including tumor cells, immune cells, fibroblasts, endothelial cells, and others. These cells interact through complex signaling networks, collectively influencing tumor progression, metastasis, and therapeutic response [3,4]. However, traditional bulk transcriptome sequencing techniques are unable to accurately resolve the heterogeneity and spatial distribution characteristics of individual cells within the TME, thereby limiting a deep understanding of the pathogenesis of CRC [5]. Therefore, it is necessary to conduct in-depth research on the TME of CRC from both cellular and spatial perspectives.

With the advancements of sequencing technologies in recent years, single-cell transcriptomics (scRNA-seq) technology can comprehensively analyze the gene expression profiles at single-cell resolution, thereby identifying cell subtypes within the TME, revealing cell state transitions, and analyzing intercellular communication networks. This provides an unprecedented perspective for understanding the complexity of the TME [6,7]. Multiple single-cell studies on CRC have revealed key features of the immune microenvironment. For instance, Lee et al. have utilized scRNA-seq to demonstrate how lineage-dependent gene expression programs shape the immune landscape and identify tumor-associated cell subpopulations [8]. Zhang et al. have revealed the interactions between myeloid cells, T cells, and the stroma within CRC, thereby elucidating the mechanisms underlying myeloid-targeted immunotherapies [9]. Through scRNA-seq, Nie et al. have discovered various tumor cell subpopulations and revealed HMGA1 as a viable target for immunotherapy [10]. However, although scRNA-seq technology has successfully enabled the inference of cell–cell interaction networks and subpopulation characteristics in CRC research, single-cell sequencing inevitably loses information about the spatial localization of cells within the original CRC tissue [11,12]. Consequently, these studies still lack the observation and validation of the actual spatial distribution of these cellular communications. Therefore, integrating spatial information is crucial for understanding direct cell–cell interactions and functional regulation [13].

Spatial transcriptomics (ST) technology enables the detection of gene expression while preserving the spatial positional information of tissues, thereby providing a crucial means for understanding the spatial distribution patterns and neighborhood interactions of cells within CRC tissues [14,15], and offering a complementary approach for elucidating the TME of CRC within its spatial environment [16]. In other tumor types, ST has demonstrated unique advantages. For example, Kim et al. dissected the genetic heterogeneity of melanoma using gene expression profiles generated via ST technology [17]. Emelie Berglund et al. utilized ST to map the spatial landscape of prostate cancer tissue, revealing gene expression gradients within the stroma adjacent to tumor regions, thereby enabling the re-stratification of the TME [18]. However, compared to studies relying solely on scRNA-seq, ST research on primary CRC remains relatively limited. In particular, studies that integrate single-cell and spatial transcriptomic data to systematically investigate intercellular communication within the native spatial microenvironment of CRC are still in the developmental stage [19,20]. In addition, mainstream ST platforms represented by Visium still face technical challenges in terms of spatial resolution, as each spot typically captures 10–200 cells [21]. This mixed-cell signal makes it difficult to directly resolve the spatial distribution of individual cell types. Therefore, we comprehensively elucidated the CRC immune signaling network by integrating multi-omics data. This integration mitigates the limitations inherent to individual data modalities, thereby enabling a deeper dissection of the cellular heterogeneity, spatial distribution characteristics, and intercellular communication patterns within the CRC TME. This will help provide new perspectives for targeted immunotherapy of CRC [15,22,23].

Macrophage migration inhibitory factor (MIF) is a pleiotropic pro-inflammatory cytokine closely associated with inflammation, immune regulation, and tumor progression. MIF signals through multiple receptors, including CD74, CD44, and CXC motif chemokine receptors (CXCR2, CXCR4 and CXCR7), enabling it to act across a wide range of cell types [24,25]. Therefore, MIF-related signaling provides a relevant framework for investigating cell–cell communication within the CRC TME.

This study aims to integrate scRNA-seq and ST data to systematically analyze the cellular composition, functional heterogeneity, intercellular communication networks, and spatial distribution characteristics of the CRC TME. First, by performing high-throughput scRNA-seq analysis on primary CRC tissues, we identified and annotated the major cell types within the TME, and conducted an in-depth analysis of the functional characteristics and heterogeneity of key cell subpopulations. Subsequently, prognostic analyses were conducted in conjunction with clinical data to identify 14 prognostic genes significantly associated with overall survival (OS), and a prognostic risk score model was constructed. Furthermore, we constructed a cell–cell interaction network through cell–cell communication analysis and systematically evaluated the activity strength and patterns of different signaling pathways involved in intercellular communication by integrating signaling pathway activity analysis, thereby revealing the critical role of the MIF signaling pathway in TME regulation and tumor progression. Concurrently, we investigated the association between this pathway and patient prognosis, discovering that its key genes are closely correlated with immune-related scores and patient outcomes. Additionally, utilizing the GDSC database, we predicted the drug sensitivity associated with the key genes of the MIF pathway to explore the potential value of their expression levels in predicting the efficacy of various anti-tumor drugs. This study demonstrates that the MIF signaling pathway exerts an immunosuppressive regulatory effect within the TME of primary CRC, offering critical insights into the complexity of the CRC TME and providing potential biomarkers and therapeutic targets for prognostic assessment, immunotherapy, and personalized precision medicine in CRC.

## 2. Materials and Methods

### 2.1. Data Sources

We downloaded scRNA-seq data for five patients with primary CRC (GSM7290763, GSM7290769, GSM7290772, GSM7290773, and GSM7290774) from the GSE231559 dataset in the GEO database (https://www.ncbi.nlm.nih.gov/geo/, accessed on 22 May 2024) [26]. To provide clinical and molecular context for this single-cell cohort, we extracted existing clinical information for these five CRC tumors from the original study’s supplementary tables; this included sample type, anatomical site, patient demographics, tumor stage at sampling, microsatellite status, and known mutation status of CRC-associated genes (Table S1). These annotations were used to interpret the inter-patient heterogeneity observed in the scRNA-seq analysis. For genes not listed in the original mutation table (including *SMAD4*), we treated the data as missing rather than inferring status from the transcriptomic data. Additionally, we downloaded spatial transcriptomics (ST) sequencing data for one primary CRC tumor tissue section (GSM7058758) in the GSE225857 dataset [27]. This ST sample was used for spatial cell-type mapping, spatial niche characterization, and local ligand–receptor communication analysis. Secondly, we downloaded 499 samples (TCGA-COAD) from the TCGA database (http://portal.gdc.cancer.gov/, accessed on 22 May 2024) via the SangerBox platform (http://vip.sangerbox.com/home.html, accessed on 22 May 2024) (comprising 458 CRC tissue samples and 41 normal colorectal tissue samples), along with clinical data for 458 CRC patients [28]. Finally, we obtained microarray gene expression data for 96 patients (comprising 286 multi-regional samples from 98 primary colorectal tumors) from the GSE241101 dataset to serve as a validation dataset for subsequent analyses [29].

### 2.2. Processing of Single-Cell RNA Sequencing Data

First, we performed preprocessing and quality filtering of the scRNA-seq data (Seurat package, version 5.2.1) [30]. Single-cell transcript expression levels are quantified based on Unique Molecular Identifier (UMI) counts to reflect the transcript abundance within each cell. Genes expressed in at least three cells and cells with a gene count exceeding 800 were retained, while cells with a mitochondrial gene expression ratio greater than 25% were excluded [26]. Ultimately, 23,614 genes and 11,156 cells were obtained for subsequent analysis. Secondly, the data were subjected to standardization and normalization. The top 2000 highly variable genes exhibiting the most significant expression variation across cells were selected. Principal Component Analysis (PCA) was performed on these cells using these highly variable genes as features. Additionally, we used Harmony to integrate data and eliminate batch effects (harmony package, version 1.2.3) [31]. Subsequently, the cells were subjected to cluster analysis, visualized using Uniform Manifold Approximation and Projection (UMAP), and clusters containing fewer than 50 cells were removed. To identify the cell types within each cluster, we performed differential expression analysis comparing each cluster against all other clusters, utilizing the FindAllMarkers function to identify marker genes for each cluster. Specifically, genes are defined as significant marker genes (Up-regulated Expressed Genes, UEGs) for a target cell cluster if their expression levels within that cluster are significantly higher than in all other cell clusters ($l{\mathrm{og}}_{2}FC>0.25$, multiple-testing-corrected p-value<0.05) and if they are expressed in at least 25% of the cells within the target cluster. Finally, cell type identification and annotation were performed using the CellMarker 2.0 (http://117.50.127.228/CellMarker/, accessed on 14 September 2024) [32] and Cell Taxonomy (https://ngdc.cncb.ac.cn/celltaxonomy/, accessed on 14 September 2024) [33] databases.

### 2.3. Processing of Spatial Transcriptomics Data

For the quality control of spatial transcriptomic data, we retained genes expressed in at least three spots and filtered for spots expressing more than 200 genes. Ultimately, 3417 high-quality sequencing spots were obtained, comprising 53,077,791 unique molecular identifiers (UMIs) and 17,943 genes. To correct for high-quality spot sequencing depth, we applied SCTransform for normalization and scaling, setting the number of highly variable features to 3000. Subsequently, we employed PCA for dimensionality reduction and inter-spot similarity comparison. Following this, we performed graph-based clustering using the top 15 principal components and visualized the identified clusters via UMAP. Given that each spot typically contains multiple cells, we utilized the aforementioned scRNA-seq data as a reference to infer the predominant cell type composition within each spot (Seurat package, version 5.2.1) [30]. Finally, we mapped these predicted cell type labels back onto the corresponding spatial in situ images of the CRC tissue sections.

### 2.4. Gene Set Variation Analysis

To assess the activity of key pathways within each cell cluster, we first obtained the 50 human hallmark pathway gene sets from the MSigDB database (http://www.gsea-msigdb.org/gsea/index.jsp, accessed on 5 March 2025) (msigdbr package, version 24.1.0). Thereafter, based on the average expression matrix of each cluster, pathway enrichment scores were calculated for each cell cluster (GSVA package, version 1.40.0) [34]. To evaluate differences in pathway activity among clusters, each cell cluster was compared with all other clusters, and differential analysis was performed on the enrichment scores (limma package, version 3.62.2). The t values generated from the differential analysis reflect the relative differences in pathway activity, where positive values indicate higher activity of a given pathway in the target cell cluster, negative values indicate lower activity, and the absolute value represents the magnitude of the difference.

### 2.5. Cell Communication Analysis

To understand intercellular interactions, we inferred the strength and direction of ligand–receptor-mediated signaling pathways based on the CellChatDB database (https://github.com/sqjin/CellChat, accessed on 20 December 2024) [35], thereby performing cell–cell communication analysis of the scRNA-seq data (CellChat package, version 1.6.1). For spatial transcriptome (ST) data, we calculated local cell–cell communication strength by incorporating the spatial proximity of cells (NICHES package, version 1.1.0) [36,37], in order to reveal the influence of the spatial environment on signal transduction. Specifically, we utilized the algorithm’s ‘NeighborhoodToCell’ analysis function to calculate the communication strength of ligand–receptor (L-R) pairs between spatially neighboring cells. Building on this, we further vectorized all spatial locations by calculating the cell type composition and interaction patterns within the neighborhood surrounding each spot. Subsequently, we applied UMAP dimensionality reduction and unsupervised clustering to these feature vectors to identify niches characterized by similar microenvironmental features, thereby revealing the specific distribution of functional spatial domains.

### 2.6. TCGA Gene Expression Data Processing and Clinical Association Analysis

First, we performed a $\log_{2}(FPKM+1)$ transformation on the RNA-seq (FPKM) expression data from the TCGA-COAD cohort and removed outliers with an average expression value of less than 1 across all samples. Then, we excluded patients with a survival time of 0 days from the clinical data of 458 patients and selected those with confirmed primary tumor type information, resulting in a dataset of 338 clinical records (comprising 287 colon adenocarcinoma (CAC) samples and 51 colon mucinous adenocarcinoma (CMAC) samples). Subsequently, for the clinical data, we divided patients into high- and low-expression groups based on the median expression of the target gene, and compared survival outcomes between the different expression groups using Kaplan–Meier survival analysis (survival package, version 3.8.3). Concurrently, univariate Cox proportional hazards models were used to calculate hazard ratios (HR) and their 95% confidence intervals (CI) (forestplot package, version 3.1.7).

### 2.7. Correlation Analysis of Tumor Microenvironment Scores and Pathway Genes

To evaluate the immune and stromal components of the CRC tumor microenvironment in the TCGA-COAD cohort, we calculated the ImmuneScore, StromalScore, and ESTIMATEScore using the ESTIMATE algorithm (ESTIMATE package, version 1.0.13). Specifically, we employed the estimateScore function to infer tumor microenvironment scores from bulk transcriptomic data based on predefined immune- and stromal-related gene signatures. The ImmuneScore reflects the enrichment of immune cell-related gene signatures, whereas the StromalScore reflects the enrichment of stromal cell-related gene signatures. The ESTIMATEScore summarizes the combined enrichment of immune and stromal components within the tumor microenvironment. Higher scores indicate a greater inferred enrichment of the corresponding immune or stromal components. Subsequently, Spearman correlation analysis [38] was used to assess the associations between the expression levels of MIF pathway genes and these tumor microenvironment scores.

### 2.8. Drug Sensitivity Prediction

To assess the association between key gene expression levels and drug sensitivity, we utilized the Genomics of Drug Sensitivity in Cancer (GDSC) database (https://www.cancerrxgene.org/, accessed on 6 October 2025) to conduct a comprehensive evaluation. We utilized the R software (version 4.4.2) to construct a ridge regression model (oncoPredict package, version 1.2) [39] to predict the half-maximal inhibitory concentration (IC50) of each sample in the TCGA-COAD cohort for various drugs. We used the Wilcoxon rank-sum test to compare differences in IC50 values between different sample groups, setting a threshold of p<0.01 for statistical significance, and identified drugs showing significant differences.

### 2.9. Validation Dataset Analysis

To further validate the relationship between pathway-associated genes and immune infiltration, we utilized the GSE241101 dataset as a validation set. First, probe annotation was performed based on the platform annotation file (GPL33696), and unannotated and duplicate genes were removed (limma package, version 3.62.2). Next, the org.Hs.eg.db database (https://bioconductor.org/packages/org.Hs.eg.db/, accessed on 3 June 2025) was utilized to correct gene symbols, yielding a standardized expression matrix with gene names as row names. Finally, utilizing this validation set, we employed the same analytical methods as those applied to the TCGA-COAD cohort to calculate pathway activity scores and immune infiltration scores for each sample. We then verified the association between these two metrics using Spearman correlation analysis and assessed the relationship between pathway-related genes and clinical characteristics to validate the robustness of their clinical significance.

## 3. Results

### 3.1. Single-Cell Analysis and Cell Annotation of CRC

**Figure 1:**
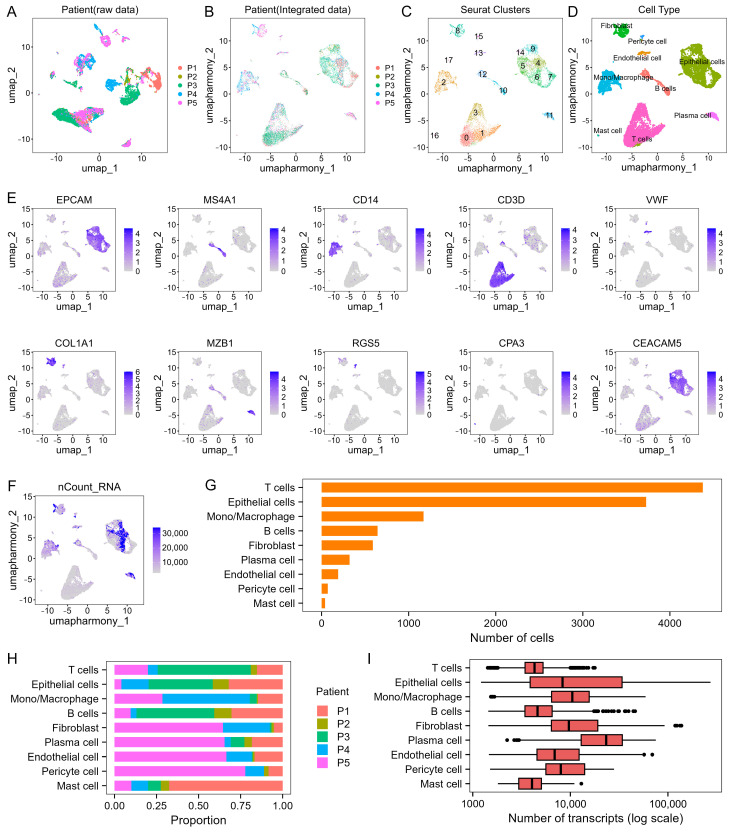
Overview of 11,156 single cells from five primary CRC samples in GSE231559. (**A**) UMAP distribution of 11,156 cells before batch correction, colored by sample origin. (**B**) UMAP distribution after Harmony-based batch correction. (**C**) UMAP distribution plot of the clustering results. (**D**) Annotation of major cell types based on canonical marker genes. (**E**) Expression patterns of representative marker genes used for cell-type annotation. (**F**) The number of transcripts (UMIs) detected in the cell clusters. (**G**) Statistical counts of each cell type across the 5 samples. (**H**) Percentage distribution of the nine annotated cell types across the five samples. (**I**) Box plots showing the distribution of transcript counts for each cell population.

To elucidate the TME of CRC, we first performed scRNA-seq analysis on primary CRC tissues. Initially, we performed dimensionality reduction and preliminary clustering on all cells. The results showed that cells from different patients were clearly separated according to their sample origin (Figure 1A). This indicates the presence of a significant batch effect among samples. To eliminate this technical bias, we used ‘Harmony’ to mitigate the impact of batch effects. The corrected results showed that cells derived from different patients were thoroughly mixed (Figure 1B). To clearly delineate the categories of these cells, we subsequently performed further clustering, which identified 18 clusters (Figure 1C). We then annotated each cell type based on the significant marker genes of each cell cluster, and the results showed that these cells were mainly classified into nine known cell lineages. Specifically, these include epithelial cells (*EPCAM*), B cells (*MS4A1*), monocytes/macrophages (*CD14*), T cells (*CD3D*), endothelial cells (*VWF*), fibroblasts (*COL1A1*), plasma cells (*MZB1*), pericytes (*RGS5*), and mast cells (*CPA3*) (Figure 1D,E). To further investigate the cellular origins of malignant cells in primary CRC, we performed a gene expression visualization analysis of representative marker genes for each cell type, as well as the known CRC marker gene *CEACAM5* [40]. The results showed that *CEACAM5* was predominantly expressed within epithelial cell clusters, suggesting that these clusters may be enriched with malignant tumor cells (Figure 1E). Furthermore, we analyzed the number of transcripts within the cell clusters. The results revealed that the UMI counts in the tumor epithelial cell clusters were significantly higher than those in the other clusters (Figure 1F). This is consistent with the generally higher transcriptional activity of tumor cells, suggesting that CRC cells may predominantly originate from epithelial cells. Finally, statistical analysis of the cell counts in CRC revealed that the numbers of T cells and epithelial cells were significantly higher than those of other cell types, indicating that they are the predominant cell types within the TME (Figure 1G). Notably, the proportions of cell types also exhibit certain variations across different samples (Figure 1H), indicating that the CRC TME displays significant inter-patient heterogeneity. In addition, to assess the transcriptional status within each subpopulation, we analyzed the distribution characteristics of transcript abundance in each cell population. The results indicate that the distribution of transcript counts in epithelial cells is relatively broad (Figure 1I), suggesting heterogeneity in transcriptional activity within the tumor epithelial cell population.

Based on the observed differences in cellular composition and transcriptional states among the samples, we integrated clinical data and mutation status annotations for CRC driver genes to interpret inter-patient heterogeneity (Table S1). All five samples are CRC tumors originating from the colon. Four samples (C1/GSM7290763, C2/GSM7290769, C3/GSM7290772, and C5/GSM7290774) are classified as stage IV MSS (microsatellite stable) tumors, while sample C4/GSM7290773 is a stage II MSI (microsatellite instability) tumor. Among the samples with available mutation annotations, *APC* is wild-type in all cases. *TP53* is mutated in samples C1–C3 but wild-type in sample C5, and *KRAS* is mutated in samples C3 and C5. These clinical and molecular differences indicate that the cohort primarily represents advanced-stage MSS colorectal cancer, alongside one early-stage MSI case, thereby providing the necessary clinical and molecular context for subsequently interpreting inter-patient transcriptional heterogeneity and intercellular signaling differences.

### 3.2. Tumor-Associated Fibroblast Subclusters

**Figure 2:**
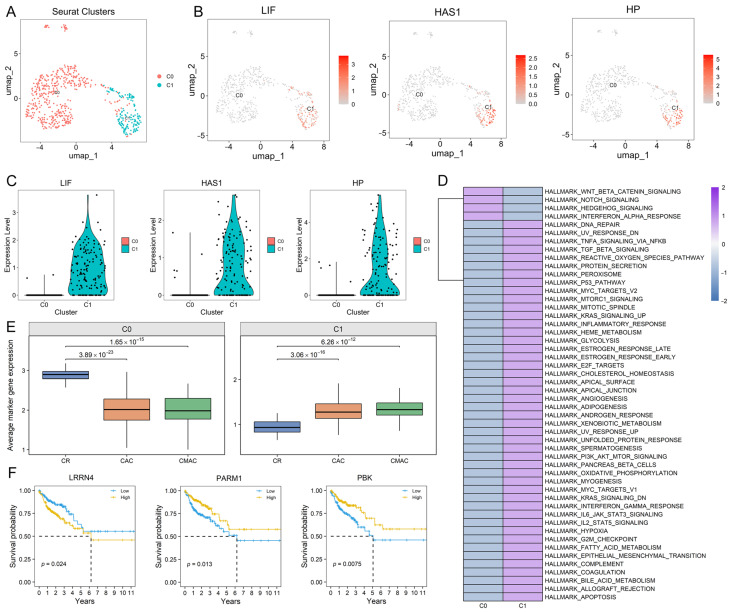
Identification and characterization of fibroblast subpopulations in the CRC scRNA-seq cohort. (**A**) UMAP distribution map of 589 fibroblasts. (**B**) UMAP feature plots of spatial expression patterns for the fibroblast marker genes *LIF*, *HAS1*, and *HP* in the single-cell embedding. (**C**) Expression levels of *LIF*, *HAS1*, and *HP* in the two fibroblast clusters. (**D**) Heatmap of GSVA pathway activity scores across different clusters. The figure displays the relative differences in pathway activity, represented as t values. (**E**) Expression levels of two fibroblast cluster marker genes across different tissue subtypes (CR: Colorectal Normal Tissue, *n* = 41; CAC: Colon Adenocarcinoma, *n* = 287; CMAC: Colon Mucinous Adenocarcinoma, *n* = 51) within the TCGA-COAD cohort. (**F**) Kaplan–Meier survival curves for high- and low-expression groups of the three marker genes associated with overall survival in CRC patients within TCGA-COAD. (Note: “C0” and “C1” labeled in the figure refer to fibroblast subcluster 0 and subcluster 1, respectively.)

Cancer-associated fibroblasts (CAFs) are one of the key components of stromal cells and play a significant role in CRC progression. To investigate their heterogeneity within CRC tissues, we performed a secondary clustering analysis on the 589 fibroblasts previously identified. Based on the expression patterns of highly variable genes, these fibroblasts are classified into two subclusters (Figure 2A). To clarify the functional characteristics of the two subclusters, we first analyzed the characteristic marker genes of each subclusters (Table S2) and found that genes such as *LIF*, *HAS1*, and *HP* were specifically highly expressed in cluster 1, which suggests that they may be closely related to promoting the functions of invasive TME, inflammatory response, and immune regulation [41,42,43,44] (Figure 2B,C). To further investigate the pathway functions of different tumor cell clusters, we performed gene set variation analysis (GSVA) to assess the differences in the activity of 50 cell function-related signaling pathways in two subclusters. The results showed that there were significant differences in pathway activity between the two subclusters, with Subcluster 1 participating in more pathways than Subcluster 0. Subcluster 0 is primarily enriched in pathways related to cellular signaling regulation (WNT_BETA_CATENIN_SIGNALING, NOTCH_SIGNALING, and HEDGEHOG_SIGNALING), whereas Subcluster 1 is broadly enriched in pathways such as epithelial–mesenchymal transition (EPITHELIAL_MESENCHYMAL_TRANSITION; EMT), inflammatory response (INFLAMMATORY_RESPONSE), TGF-β signaling (TGF_BETA_SIGNALING), TNF-α signaling via NF-κB (TNFA_SIGNALING_VIA_NFKB), glycolysis (GLYCOLYSIS), and G2/M checkpoint (G2M_CHECKPOINT), indicating that Subcluster 1 possesses stronger immune response capacity, metabolic activity, and mesenchymal transition capacity. These characteristics are highly consistent with the related functions of CAFs in promoting tumor progression [45,46,47] (Figure 2D). Subsequently, to validate the relevance of the aforementioned characteristics of the two subpopulations in clinical samples, we analyzed the expression levels of the top 30 significant marker genes for each subpopulation in normal colorectal (CR) tissue, colon adenocarcinoma (CAC), and colon mucinous adenocarcinoma (CMAC) within the TCGA-COAD dataset. The results showed that the expression levels of marker genes for Subcluster 0 were significantly lower in tumor tissues (CAC and CMAC) compared to CR tissues, whereas the expression levels of marker genes for Subcluster 1 were significantly upregulated in CAC and CMAC, exhibiting a contrasting expression trend (Figure 2E). The upregulation of subcluster-1-related genes suggests that they may be involved in the stromal remodeling process that promotes tumorigenesis and progression, while subcluster-0-related genes are mainly expressed in normal tissues and are associated with homeostatic stromal function.

Given that Subcluster 1 exhibits distinct pro-tumor characteristics, we further investigated the association between its marker genes and the prognosis of COAD patients. Specifically, we utilized the signature genes of two fibroblast subpopulations as a candidate gene set and conducted a survival analysis by integrating clinical follow-up data from the TCGA-COAD cohort. First, single-factor Cox proportional hazards models were utilized to assess the survival association of each gene, identifying three genes (*LRRN4*, *PARM1*, and *PBK*) significantly associated with the overall survival of CRC patients (p<0.05). Subsequently, patients were classified into high- and low-expression groups based on the median expression levels of these three genes, and Kaplan–Meier survival analysis was employed to compare the survival differences between the two groups. The results (Figure 2F) showed that patients with high expression of *LRRN4*, as well as those with low expression of *PARM1* and *PBK*, exhibited significantly reduced survival rates, suggesting that these genes may serve as potential biomarkers for CRC prognosis. Taken together, these results indicate the presence of functionally heterogeneous CAF subpopulations in CRC. Subpopulation 0 is primarily characterized by an enrichment of signaling pathways and may play a fundamental role in regulating signaling networks within the TME, whereas subpopulation 1 exhibits stronger pro-tumor characteristics through the overexpression of inflammation-related genes and the activation of various pro-tumor signaling pathways, such as EMT and TGF-β, and may thus play a significant pro-tumor role in the progression of CRC.

### 3.3. Distinct Epithelial Cell Subclusters Exist in Primary CRC Tumors

**Figure 3:**
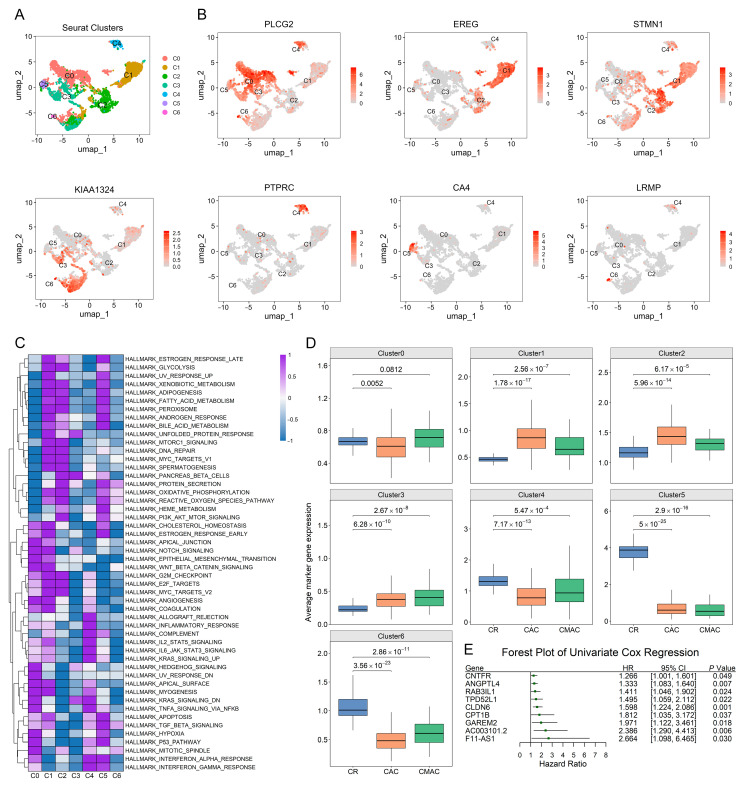
Identification and characterization of epithelial cell subpopulations in the CRC scRNA-seq cohort. (**A**) UMAP distribution map of 3728 epithelial cells. (**B**) Expression patterns of representative marker genes across epithelial cell subpopulations. (**C**) Pathway activity based on GSVA scores among different clusters. The color gradient in the figure represents the t values of relative differences in pathway activity. (**D**) Expression of epithelial cell marker genes within each cluster in normal colorectal tissue (CR, *n* = 41), colon adenocarcinoma (CAC, *n* = 287), and colon mucinous adenocarcinoma (CMAC, *n* = 51). (**E**) Forest plot of univariate Cox regression results for epithelial marker genes significantly associated with overall survival in CRC patients from TCGA-COAD. (Note: The labels “C0” through “C6” in the figure refer to epithelial cell subcluster 0 through 6, respectively.)

Epithelial cells are the main tumor cell component of CRC, and their heterogeneity is closely related to the malignancy of the tumor and the treatment response. To deeply investigate the heterogeneity of epithelial cells within CRC tissues, we re-clustered the 3728 epithelial cells previously identified. Based on the expression patterns of highly variable genes, a total of 7 subclusters were obtained (Figure 3A). The results demonstrate that the various subclusters are clearly distributed with distinct boundaries within the reduced-dimensional space, indicating the presence of significant heterogeneity among these subclusters. To elucidate the molecular characteristics of distinct epithelial cell subpopulations, we performed differential gene expression analysis on each of the seven subpopulations and identified characteristic marker genes for each group (Table S3). The expression patterns of selected representative marker genes across the subclusters indicate that *PLCG2* is highly expressed primarily in subcluster 0, *EREG* is enriched in subcluster 1, *STMN1* is enriched in subcluster 2, *KIAA1324* marks subcluster 3, *PTPRC* is enriched in subcluster 4, *CA4* marks subcluster 5, and *LRMP* specifically marks subcluster 6 (Figure 3B). The distribution patterns of these marker genes validate the accuracy of the sub-cluster partitioning and provide a molecular basis for subsequent functional analysis. To systematically characterize the functional states of the various subpopulations, we compared the activity of each cluster across 50 cell function-related signaling pathways. The results revealed functional diversity among the epithelial cell subpopulations (Figure 3C). First, subclusters 0 and 1 collectively represent a malignant phenotype characterized by high invasiveness and the capacity for microenvironmental remodeling. Both of these subclusters are significantly enriched in pathways associated with epithelial–mesenchymal transition (EMT) and coagulation. Such subclusters of tumor epithelial cells often serve as the primary driving forces behind local tumor invasion, stromal network remodeling, and the promotion of distant metastasis [48]. Despite sharing the above commonalities, subcluster 0 specifically exhibits synergistic activation of development-related signaling pathways such as WNT_BETA_CATENIN_SIGNALING, HEDGEHOG_SIGNALING, and NOTCH_SIGNALING, suggesting that it possesses strong cellular plasticity potential. Secondly, subcluster 2 exhibits a relatively pure, pro-tumorigenic, and highly proliferative profile; it demonstrates high levels of enrichment primarily within the E2F_TARGETS, G2M_CHECKPOINT, MYC_TARGETS_V1, and MYC_TARGETS_V2 pathways, yet displays relatively low activity in invasive pathways such as EMT and coagulation. This subcluster may represent a rapidly expanding cell population within the main tumor mass. Third, subcluster 4 was significantly enriched in immune–inflammatory pathways (TNFA_SIGNALING_VIA_NFKB, IL2_STAT5_SIGNALING, IL6_JAK_STAT3_SIGNALING, etc.), whereas proliferation-related pathways were significantly suppressed. This unique molecular phenotype closely aligns with the cellular characteristics of the inflammation-induced Senescence-Associated Secretory Phenotype (SASP) within the TME and may play a pivotal role in mediating local tumor immune heterogeneity and immune evasion [49]. The remaining subclusters, meanwhile, reflect specific stress and metabolic states within the TME. Subcluster 5 is significantly enriched in the hypoxia (HYPOXIA), p53, and apoptosis (APOPTOSIS) pathways, which may represent a population of tumor epithelial cells under hypoxic stress. Subcluster 3 is specifically enriched in endocrine (PANCREAS_BETA_CELLS) and metabolic-related pathways, while subcluster 6 has the lowest overall activity in these pathways.

Furthermore, to validate the expression patterns of the aforementioned seven subclusters in clinical samples, we analyzed the expression levels of marker genes for the different subclusters in normal colorectal (CR), colon adenocarcinoma (CAC), and colon mucinous adenocarcinoma (CMAC) tissues within the TCGA-COAD dataset (Figure 3D). The results indicate that the marker genes of Subcluster 0 exhibit a significant difference between CR and CAC (p=0.0052), yet show no significant difference between CR and CMAC (p=0.0812), suggesting that this subcluster possesses adenocarcinoma subtype specificity. The expression levels of marker genes in subclusters 1, 2, and 3 were higher in tumor tissues (CAC and CMAC) than in normal colorectal (CR) tissues, consistent with their high proliferative and secretory characteristics observed in GSVA, suggesting that they may play a promoting role in tumorigenesis and progression. Conversely, the marker genes of subclusters 4, 5, and 6 were all downregulated in tumors. Combined with the functional analysis results, subcluster 4 was enriched in immune–inflammatory pathways but downregulated in tumors, which may reflect that tumors achieve immune escape by inhibiting the immune response of epithelial cells. However, the downregulation observed in tumors of subclusters 5 and 6 may reflect the loss of functional epithelial cells during the process of tumor dedifferentiation. Given that certain subclusters exhibit distinct pro-tumor characteristics, we further investigated the association between their marker genes and the prognosis of COAD patients. Specifically, we extracted the top 30 significant marker genes from each subcluster identified in the differential expression analysis of epithelial cells and performed univariate Cox regression analysis on patient survival based on the TCGA-COAD cohort. The results showed that nine genes were significantly associated with CRC survival (p<0.05), and all served as risk factors (HR>1) (Figure 3E). These genes mainly originate from subclusters 0, 1, and 2. Among them, genes from subcluster 1 (*AC003101.2*, *CLDN6*) are active in cell proliferation-related pathways, *CNTFR* originates from subcluster 5, and *F11-AS1* originates from subcluster 3. These findings suggest that epithelial cell subpopulations in distinct functional states influence the prognosis of CRC patients through different mechanisms and also provide important clues for understanding the mechanisms of tumorigenesis.

### 3.4. T-Cell Subclusters

**Figure 4:**
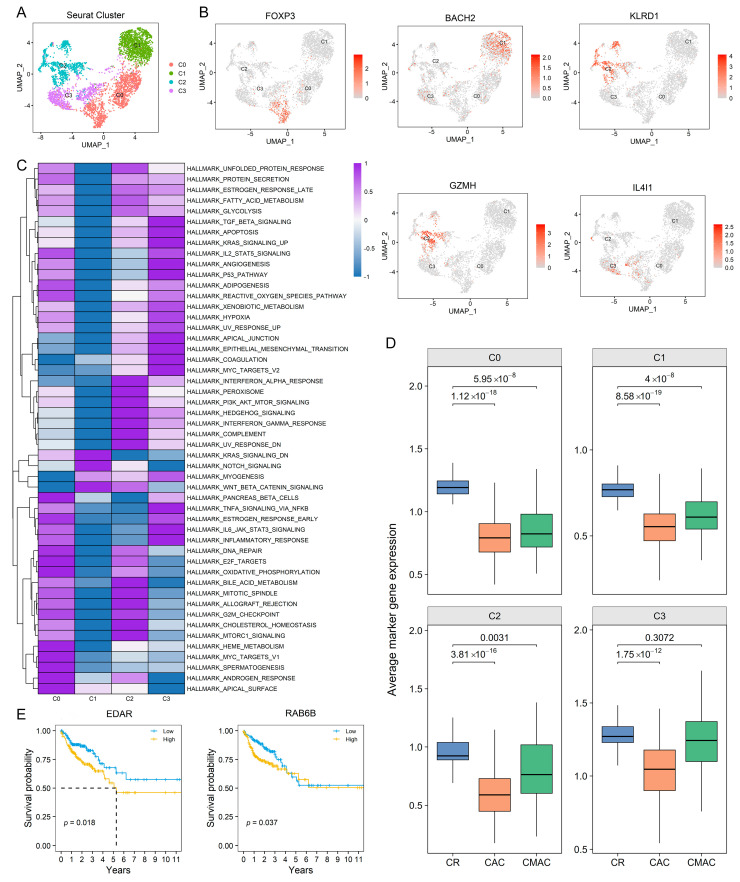
Identification and characterization of T-cell subpopulations in the CRC scRNA-seq cohort. (**A**) UMAP distribution of 4377 T cells. (**B**) Expression patterns of representative marker genes across T-cell subclusters. (**C**) GSVA-based pathway activity scores across T-cell subclusters. The color gradient represents the t values reflecting relative differences in pathway activity. (**D**) Expression of T-cell marker genes from each cluster in normal colorectal tissue (CR, *n* = 41), colonic adenocarcinoma (CAC, *n* = 287), and colonic mucinous adenocarcinoma (CMAC, *n* = 51) samples from TCGA-COAD. (**E**) Expression and survival association of two T-cell marker genes associated with overall survival in CRC patients from TCGA-COAD. (Note: The labels “C0” through “C3” in the figure refer to T-cell subclusters 0 through 3, respectively.)

T cells are key immune cells within the TME, and their functional state directly influences anti-tumor immune responses and patient prognosis. To systematically dissect the functional heterogeneity of T cells within the CRC microenvironment, we performed a secondary clustering analysis on the 4377 T cells previously identified. These cells were classified into four functional subclusters (Figure 4A). To elucidate the functional characteristics of each subcluster, identification was performed based on marker genes derived from differential expression analysis of each subcluster (Table S4), in conjunction with the expression distribution patterns of classic marker genes reported in previous studies. The results showed that subset 0 significantly overexpressed the *FOXP3* gene, which encodes the core transcription factor of Treg cells, suggesting that it mainly consists of regulatory T cells (Tregs) [50]. Subcluster 1 highly expressed the gene *BACH2*, which is associated with memory T-cell differentiation, suggesting that it may represent memory T cells [51,52]. Subcluster 2 significantly overexpressed the genes *KLRD1* and *GZMH*, which encode cytotoxic effector molecules, suggesting that it may represent cytotoxic T cells with killing functions (such as effector CD8+ T cells or mature NKT cells) [53]. Subcluster 3 specifically overexpressed the gene *IL4I1*, which encodes a metabolic enzyme (Figure 4B); therefore, it may represent a population of T cells in an exhausted state capable of inducing immune tolerance in the microenvironment via metabolic pathways [54]. To systematically compare the functional states of different T-cell clusters, we performed Gene Set Variation Analysis (GSVA). The results revealed significant differences between Subpopulation 1 and the other three subpopulations (Figure 4C). Among them, subcluster 0 is enriched in activation-related pathways such as SPERMATOGENESIS and MYC_TARGETS_V1, suggesting that although this subcluster possesses immunosuppressive functions, it also exhibits relatively high metabolic and transcriptional activity, which is consistent with the characteristic that Tregs require high metabolic activity to maintain their suppressive functions. The enrichment pattern of the subcluster 1 pathway suggests that the cells may be in a certain state of differentiation. Subcluster 2 was significantly enriched in immune activation pathways such as INTERFERON_ALPHA_RESPONSE and COMPLEMENT, suggesting that this subgroup consists of activated effector T cells. Subcluster 3 is significantly enriched in the TGF-β signaling pathway (TGF_BETA_SIGNALING). As TGF-β is a key immunosuppressive factor, this finding aligns strongly with the assessment that this subcluster likely consists of T cells in an immunosuppressive or functionally exhausted state. Subsequently, to assess the expression patterns of T-cell subset characteristics in clinical samples, we utilized the TCGA-COAD dataset to analyze the differential expression of the top 30 significant marker genes within each subset between tumor and normal tissues. We found that the marker genes for all clusters exhibited higher expression levels in normal colorectal (CR) tissue than in CAC and CMAC (Figure 4D). These results suggest that in the TME, the transcriptional program of these cell subclusters is significantly suppressed, and transcriptional activity is generally reduced. Furthermore, to investigate the association between T-cell subset marker genes and the prognosis of COAD patients, we conducted a survival analysis incorporating these marker genes. The results revealed that the *EDAR* and *RAB6B* genes were significantly associated with CRC survival rates (p<0.05) (Figure 4E), suggesting that these T-cell-associated genes may possess prognostic predictive value. Collectively, these results reveal the functional diversity of T cells within the CRC TME, suggesting that different subclusters may play distinct roles in anti-tumor immunity and immunosuppression.

### 3.5. Analysis of Intercellular Communication Based on Single-Cell Transcriptomics

**Figure 5:**
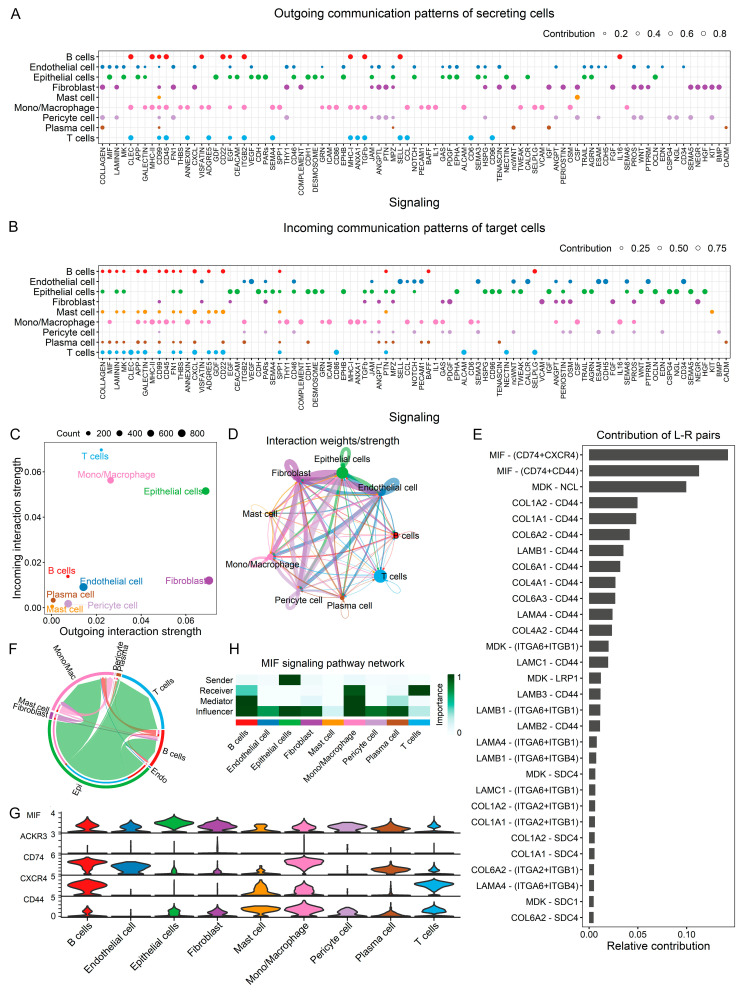
Cell–cell communication network in the CRC microenvironment inferred from scRNA-seq data. Cell–cell communication analysis was performed using CellChat based on annotated cell populations from five primary CRC samples. (**A**) Comparison of the signal output (sending) patterns of various cell populations across different signaling pathways (ordered by intensity from left to right). (**B**) Comparison of signal reception patterns across different signaling pathways for each cell type (ordered by intensity from left to right). (**C**) Strengths of outgoing and incoming interactions for each cell type. (**D**) Global cell–cell communication network showing the overall interaction weights among major cell types across all inferred signaling pathways. Circle size represents the number of interactions, and line thickness represents interaction strength. (**E**) Relative contributions of cell-specific ligand–receptor pairs within selected signaling pathways. (**F**) Chord diagram illustrating the direction of communication flow and interaction strengths of MIF signaling between different cell clusters. (**G**) Violin plots showing the gene expression levels of ligands and receptors involved in the MIF signaling network. (**H**) Relative importance scores of different cell types in functional roles within the MIF signaling pathway.

Intercellular communication is a key mechanism in shaping the TME and regulating tumor progression. To systematically dissect the interactions among different cell types within the CRC TME, we investigated the communication patterns among the nine major cell types previously identified. First, we conducted a panoramic analysis of the overall cellular communication network. The results revealed that nine cell types communicate via 89 pathways. Distinct cell types exhibited significant differences in both the number and intensity of pathway engagement, suggesting the presence of a complex intercellular communication network within the CRC microenvironment (Figure 5A,B). Furthermore, to identify the cell types playing a pivotal role within the complex network, we evaluated the overall communication characteristics of each cell type, including signal sending strength and signal receiving strength (Figure 5C). Analysis of intercellular interaction strength reveals that fibroblasts possess the strongest signal output capacity, indicating that they serve as the primary source of secreted signals within the microenvironment. T cells and monocytes/macrophages showed strong signal reception capabilities, suggesting that these immune cells mainly act as signal responders. Epithelial cells, in contrast, exhibited high levels of both signaling output and reception, suggesting that they may act as communication hubs within the TME, capable of transmitting signals to surrounding cells as well as receiving and responding to microenvironmental signals. The intercellular interaction network further visualized the dense interaction connections among various cell types (Figure 5D and Figure S1), wherein extensive communication links were established among epithelial cells, fibroblasts, and immune cell populations (including B cells, mast cells, monocytes/macrophages, plasma cells, and T cells). This formed a communication network centered on tumor cells (primarily epithelial cells) and stromal cells (comprising fibroblasts, endothelial cells, and pericytes), with broad participation from immune cells. Based on this finding, we further ranked the contributions of specific ligand–receptor pairs. The results showed that the MIF-(CD74+CXCR4) and MIF-(CD74+CD44) complex receptor signals in the MIF pathway ranked first and second in terms of contribution among all ligand–receptor pairs and significantly outperformed other communication pairs, indicating that MIF signaling has a potential core role in intercellular communication in the TME (Figure 5E). This finding suggests that the MIF signaling pathway may serve as a key hub for coordinating the functions of various cell types within the TME.

Therefore, we next focus on the MIF signaling pathway to analyze its specific modes of cellular communication. First, we visualize the direction and strength of MIF signaling transmission between different cell types using a chord diagram (Figure 5F). The results clearly reveal the unidirectional nature of MIF signaling, wherein epithelial cells serve as the primary senders of MIF signals, establishing extensive communicative connections with various types of immune cells and thereby forming a dominant, unidirectional communication pattern flowing from tumor/stromal cells to immune cells. This direction of communication suggests that tumor cells and stromal cells may actively modulate the functional state of immune cells through the secretion of MIF. To validate the molecular basis of this communication mode, we analyzed the expression distribution of the MIF ligand and its receptor molecules across different cell types. The results showed that MIF ligands were mainly highly expressed in epithelial cells and fibroblasts, while the main receptor molecules (CD74, CXCR4, and CD44) were mainly enriched in immune cell populations (B cells, mast cells, monocytes/macrophages, plasma cells, and T cells) (Figure 5G). We further utilized CellChat’s signaling network functional role analysis to comprehensively assess the roles played by each cell type within the MIF pathway (Figure 5H). The results indicate that while immune cells primarily function as signal receivers, B cells and monocytes/macrophages also serve as important mediators and influencers. This suggests that the signaling is not isolated but instead regulates the TME through intercellular communication. The effects of these signaling pathways are not limited to immune cells but may also involve stromal components such as fibroblasts and endothelial cells, thereby contributing to the regulation of tumor progression.

### 3.6. Spatial Transcriptomic Analysis Reveals the Spatial Heterogeneity of the CRC Tumor Microenvironment

**Figure 6:**
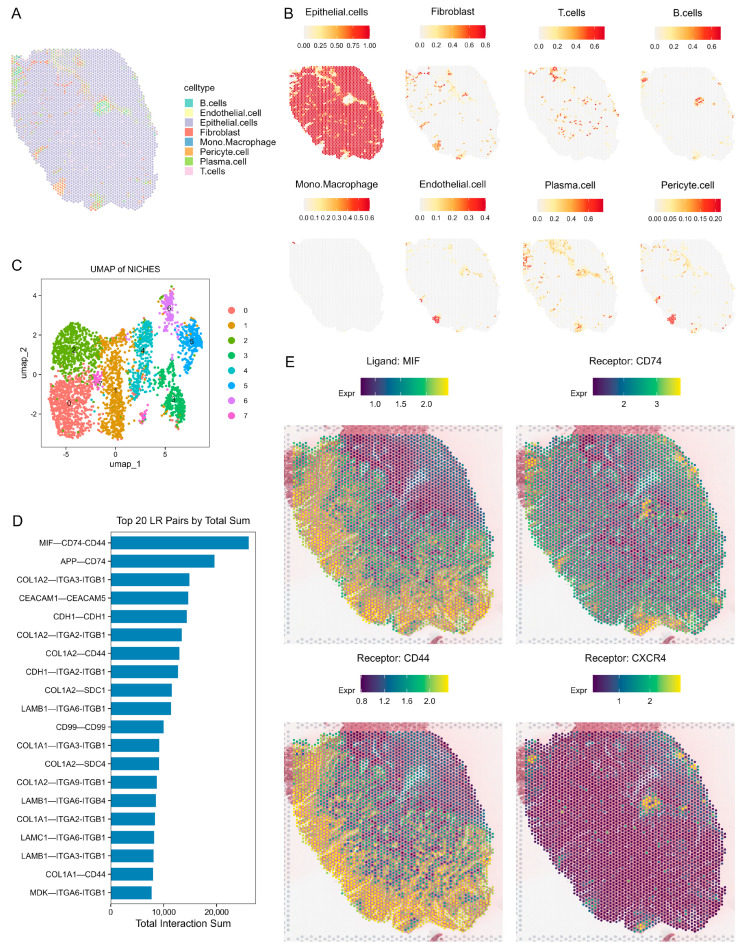
Spatial localization of cell types and MIF-related signaling in one primary CRC tissue section from GSE225857 (GSM7058758). (**A**) Spatial scatter pie chart showing the predicted proportions of major cell types at each capture spot of the CRC tissue section. (**B**) Relative abundance of each cell type in the spatial transcriptomic tissue section. (**C**) Using the NICHES method, local cell–cell communication was inferred by restricting candidate interactions to spatially proximal regions. Unsupervised clustering of spot-level communication profiles identified eight spatial niches, visualized by UMAP. Different colors represent distinct niche clusters. (**D**) The overall strength of ligand–receptor interactions between the top 20 spatially proximal cells identified in the NICHES analysis. (**E**) Spatial expression and colocalization profiles of key genes in the MIF signaling pathway within the primary CRC tissues section in situ.

While the preceding scRNA-seq analysis revealed the cell types and communication networks within CRC, it lacked information regarding the spatial localization of these cells within the tissue. To validate the activity patterns of the MIF signaling pathway at the spatial level and to elucidate the spatial distribution characteristics of primary CRC, we integrated scRNA-seq and ST data for analysis. First, to reconstruct the spatial distribution of cell types, we utilized cell types identified via scRNA-seq as a reference to predict the cellular composition of each spatial spot, subsequently mapping these predictions onto the spatial coordinates of the CRC tissue sections. Spatial scatter pie plots illustrated the spatial distribution proportions and characteristics of eight cell types within the tissue. Epithelial cells dominated in the tumor area, whereas immune cells such as T cells and monocytes/macrophages were mainly distributed in the tumor–stroma interface and stromal regions (Figure 6A,B). The spatial partitioning patterns of these cell types reveal the heterogeneity of the CRC microenvironment at the tissue structural level. To further elucidate the functional spatial organization of the TME, we analyzed the communication patterns among spatially proximal cells. The results showed that the spatial sites were divided into 8 niche clusters. In the UMAP dimensionality reduction space, these niches exhibited a distribution feature of close proximity and overlapping edges, suggesting that different niches have their own specificity in communication patterns and a certain degree of functional association, reflecting the significant spatial heterogeneity of the CRC TME at the functional level (Figure 6C). Upon obtaining spatial niche information, and to validate the communicative activity within the spatial microenvironment of the key ligand–receptor pair (MIF signaling pathway) identified in the preceding single-cell analysis, we calculated the communication strength of ligand–receptor (L-R) pairs between spatially proximal cells and ranked them based on their total spatial communication strength within the spatial microenvironment (Table S5). The results showed that, among the top 20 L-R pairs, the MIF-(CD74+CD44) complex signal ranked first, and its spatial communication strength was significantly higher than that of other ligand–receptor pairs (Figure 6D). This finding provides spatial validation of the key cellular signaling pathways identified in single-cell analyses (namely, the MIF-centered ligand–receptor interaction network) and their high level of activity. It further confirms that MIF exerts strong communication functions in the spatial microenvironment of CRC through the formation of complexes with its primary receptor CD74 and co-receptor CD44, providing important insights into the regulatory mechanisms of the MIF signaling pathway in the spatial TME of CRC [55]. Notably, although the MIF-(CD74+CXCR4) signaling complex ranked first in terms of contribution in single-cell analysis, its ranking was relatively lower in spatial analysis. This discrepancy highlights the limitations of inferring cell–cell communication solely from scRNA-seq data, which primarily calculates theoretical communication probabilities based on the average expression levels of ligands and receptors across different cell populations, whilst overlooking the spatial barriers present in the real-world tissue microenvironment [56,57]. The low activity of this composite signal in situ precisely reflects the significant spatial isolation between immune cells expressing CXCR4 and the tumor core region with high MIF expression. This indicates that although the complex receptor theoretically possesses extremely high communication activity, its actual range of action in situ is relatively limited due to the spatial structural constraints of the CRC TME [58,59]. Finally, to gain a deeper understanding of the molecular basis of the MIF signaling pathway in the spatial microenvironment, we plotted the spatial expression maps of key genes of the two core composite signals of the MIF signaling pathway (namely, the core signaling axis ligand MIF and the receptors CD74, CD44 or CXCR4) (Figure 6E). Spatial characteristics showed that MIF ligands were widely and strongly expressed in CRC tissues, with high expression signals mainly covering epithelial cells in the tumor core area, while expression was extremely low in immune and stromal enrichment areas. As the main receptor for MIF, CD74 is widely distributed in tissues and shows a significantly high expression signal in some immune-enriched areas with low MIF expression, exhibiting obvious spatial complementarity of ligand and receptor distribution characteristics. This suggests that the MIF signal may be directionally transmitted from the tumor core to the surrounding immune cell-enriched areas in a paracrine manner. At the co-receptor level, CD44 and CXCR4 exhibit distinctly different spatial distribution patterns. Regions of high CD44 expression exhibit substantial overlap with MIF, with both being concentrated within the tumor core. In contrast, the overall expression level of CXCR4 is relatively low, and its distribution is rather restricted; its colocalization with MIF is largely confined to specific spatial microdomains, suggesting that the MIF-(CD74+CXCR4) signaling complex may exert its effects within specific regions of the immune microenvironment. In summary, we systematically validated the important role of the MIF signaling pathway in the CRC microenvironment from a spatial perspective. This spatial heterogeneity and functional stratification of ligand and receptor expression not only validated the findings in single-cell analysis regarding MIF-mediated communication and interaction between tumor cells and immune cell populations in the microenvironment but also revealed, at the in situ tissue level, the spatial basis for the multifunctional role of MIF signaling within the complex TME.

### 3.7. The Immunosuppressive Environment Induced by the MIF-CD74 Pathway

**Figure 7:**
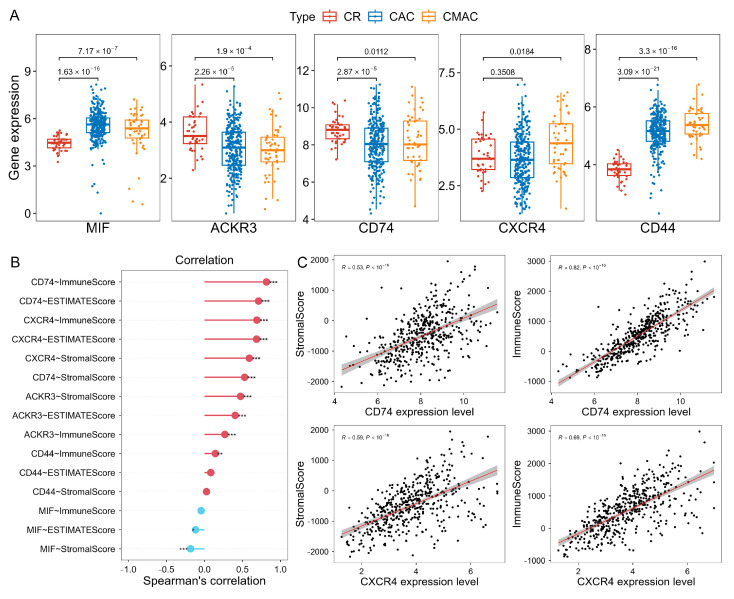
Association between the expression of five genes involved in the MIF signaling network and the tumor immune microenvironment. (**A**) Expression levels of five key MIF pathway genes in normal colorectal tissue (CR), colon adenocarcinoma (CAC), and colon mucinous adenocarcinoma (CMAC) from the TCGA-COAD database. (**B**) Spearman correlation analysis between the expression of five MIF pathway genes and tumor microenvironment scores calculated using the ESTIMATE algorithm (* means p<0.05, ** means p<0.01, *** means p<0.001). (**C**) Scatter plots showing the Spearman correlations between the expression levels of the core receptors CD74 and CXCR4, and the ImmuneScore and StromalScore.

To comprehensively investigate the mechanisms underlying the role of the MIF pathway in CRC, we analyzed the expression levels of the ligands and receptors within this pathway using gene expression data from TCGA-COAD. The results showed that all ligands and receptors exhibited significantly differential expression between CR and CAC, as well as between CR and CMAC (Figure 7A). Specifically, the expression levels of *MIF* in CAC and CMAC were significantly higher than in CR (p<0.05), suggesting that *MIF* may be aberrantly activated during the initiation and progression of CRC. At the receptor level, *ACKR3* and *CD74* exhibit significantly downregulated expression in CRC. *CXCR4* showed only slight differences between the two types of CRC and CR, which may reflect its low abundance expression in specific cell subpopulations, consistent with our previous observation of low *CXCR4* expression in spatial transcriptome analysis. *CD44* expression levels in CRC were significantly higher than those in normal tissues, which is consistent with the upregulation pattern of *MIF* (Figure 7A).

Subsequently, we further investigated the association between the MIF pathway and the tumor immune microenvironment. To this end, we calculated the ImmuneScore, StromalScore, and ESTIMATEScore for 458 CRC samples and analyzed the correlation between the expression of key genes in the MIF pathway and these scores. The results demonstrated that the expression levels of CD74, CXCR4, and ACKR3 were significantly positively correlated with the immune microenvironment score (*** means p<0.001, ** means p<0.01), suggesting that these receptors may play a pivotal role in regulating the tumor immune microenvironment. Conversely, the expression level of the ligand MIF was significantly negatively correlated with the stromal score (*** means p<0.001, * means p<0.05), leading to the speculation that it may be involved in immune-suppressive processes [60] (Figure 7B,C). Finally, to validate the reliability of the above results, we performed the same pathway gene expression analysis on the GSE241101 dataset. The validation results further confirmed the expression trends observed in the TCGA-COAD dataset (Figure S2), indicating the reliability of our results. Synthesizing these analytical results, we infer that the MIF pathway occupies a central position as primary CRC cells establish complex signaling regulatory networks to remodel the tumor immune microenvironment. Specifically, the MIF-CD74 axis serves as the primary signaling axis, with CD44 or CXCR4 serving as co-receptors to regulate immune cell responses within the TME through a composite signaling transduction mode.

### 3.8. Drug Prediction for Key Genes in the MIF Signaling Pathway

**Figure 8:**
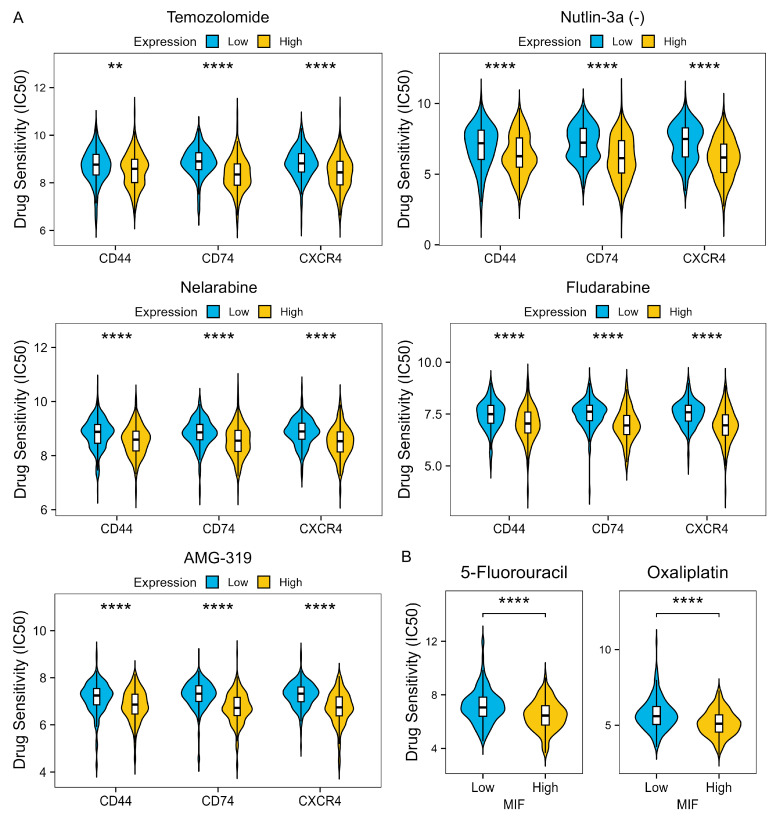
Analysis of drug sensitivity of key genes in the MIF signaling pathway. (**A**) Differences in drug sensitivity to common targeted agents and kinase inhibitors (including Temozolomide, Nutlin-3a (-), Nelarabine, Fludarabine, and AMG-319) between groups with high versus low expression of key MIF receptor complex genes (*CD44*, *CD74*, *CXCR4*) (** means p<0.01, **** means p<0.0001). (**B**) Comparison of predicted IC50 values for 5-fluorouracil and oxaliplatin between patients in the high- and low-MIF expression groups (**** means p<0.0001).

To further explore the potential clinical translational value of key genes in the MIF signaling pathway in the precision treatment of CRC, we predicted the differences in sensitivity (IC50 value) to common antitumor drugs among patients with different gene expression levels in the TCGA-COAD cohort based on the GDSC database. The results showed that patients with high expression of *CD44*, *CD74*, and *CXCR4* had significantly lower predicted IC50 values for Temozolomide, suggesting that they may have relatively higher drug sensitivity. Similarly, drugs such as the MDM2 antagonist (Nutlin-3a (-)), Nelarabine, Fludarabine, and the PI3K inhibitor (AMG-319) also demonstrated higher drug sensitivity in patients with high expression of *CD44*, *CD74*, and *CXCR4* (** means p<0.01, **** means p<0.0001) (Figure 8A). However, the prediction of drug sensitivity based on MIF exhibits relatively independent characteristics. Patients with high MIF expression had significantly lower predicted IC50 values for the drugs 5-Fluorouracil and Oxaliplatin in classic gastrointestinal chemotherapy regimens for CRC than those with low expression (p<0.0001) [61,62] (Figure 8B), suggesting a potential association between MIF expression levels and drug sensitivity to classic chemotherapy regimens for CRC.

## 4. Discussion

The intricate network of cellular interactions within the TME plays a pivotal role in tumor immune evasion and disease progression. This study systematically characterized the molecular features and cellular communication patterns of the microenvironment in primary CRC. By integrating scRNA-seq with spatial transcriptomics, we identified nine major cell types within the TME and characterized their functional heterogeneity. Secondly, it was revealed that the MIF signaling pathway (mainly the MIF-(CD74+CD44) and MIF-(CD74+CXCR4) complex signaling) is one of the important pathways mediating the interaction between tumor cells and immune cells. Furthermore, this study provides in situ spatial validation of the significant spatial heterogeneity within the ligand–receptor network and elucidates that the expression of key genes in the MIF signaling pathway is closely correlated with patients’ immune scores and prognosis. Moreover, drug sensitivity prediction analysis further suggested that the expression levels of key genes in the MIF pathway may influence patients’ responses to specific chemotherapeutic agents, thereby providing direction for future research into personalized therapeutic strategies based on the MIF pathway.

Through the analysis of scRNA-seq data from primary CRC, cells were identified as belonging to nine major cell population types, revealing the high heterogeneity of the TME. Notably, the five scRNA-seq samples exhibit variations in clinical and molecular characteristics. Four of the samples are stage IV MSS-status CRC, while C4 is a stage II MSI-status tumor. Regarding mutation profiles, samples C1–C3 harbor TP53 mutations, while C3 and C5 carry KRAS mutations. This clinical and molecular heterogeneity among patients may have influenced, to some extent, the observed differences in transcriptional status and intercellular communication. Nevertheless, an MIF-centered signaling axis was consistently identified across multiple analytical strategies, suggesting it represents a common communication pattern within the CRC tumor microenvironment rather than one restricted to a specific disease stage or molecular subtype. Furthermore, the major cell types identified in this study are largely consistent with the major cell types (covering core components such as epithelial cells, immune cells, and mesenchymal cells) identified by CRC in similar scRNA-seq studies in recent years [10,63,64], verifying the reliability of the data and the accuracy of cell annotation in this study. Based on this, we observed that various cell types exhibited significant subpopulation heterogeneity, suggesting that distinct cell subpopulations may play differentiated functional roles in tumor progression. This finding supports the notion that future therapeutic strategies should incorporate the principles of personalized precision medicine [65]. Specifically, at the epithelial cell level, the significant differentiation of subpopulations suggests that tumor epithelial cells possess a potential capacity to drive tumor invasion. At the fibroblast level, some subsets exhibit the potential to promote inflammation, immune regulation, and tumor metastasis. This is consistent with the fact that cancer-associated fibroblasts (CAFs) promote CRC metastasis through mechanisms such as EMT induction, angiogenesis promotion, ECM remodeling, and cytokine secretion, and interact with cancer cells to construct a microenvironment conducive to tumor progression [66,67,68,69,70]. At the T-cell level, the presence of regulatory T cells (Tregs) is closely associated with immune evasion in CRC [50,51]. Conversely, the coexistence of cytotoxic T cells and immunosuppressive T cells suggests that, even within an immunosuppressive microenvironment, a certain degree of anti-tumor immune potential remains. This coexisting “activation–inhibition” immune state may be an important reason for individual differences in the response to immunotherapy [71]. These results demonstrate the complexity of the TME and provide important insights for precision medicine, such as the possibility that targeting specific functional subgroups may be more effective than targeting the entire cell type in general [72,73,74].

Intercellular communication analysis reveals that the MIF signaling pathway occupies a pivotal position within the CRC TME. This finding aligns with the known function of MIF as a multifunctional inflammatory mediator, the aberrant expression of which is closely associated with immunosuppression [60,75]. Notably, epithelial cells exhibit the strongest capacity for MIF signal transmission, whereas immune cells primarily function as signal recipients, suggesting that tumor cells may actively remodel the immunosuppressive environment through the release of MIF signals. Previous studies by Xie et al. have demonstrated that MIF exerts pro-tumorigenic effects in various malignancies through immune evasion mechanisms [76], thereby supporting the potential classification of MIF as a therapeutic target for CRC patients. Previous studies have confirmed that when both CD74 and CD44 are present in CRC, the tumor becomes more aggressive [10,62,77,78], which corroborates the results of our analysis. Further analysis of the immune microenvironment revealed that MIF expression was negatively correlated with immune scores, while the expression of its receptors, CD74 and CXCR4, was positively correlated with immune scores. This suggests that MIF may promote tumor immune escape by inhibiting immune cell infiltration, and the high expression of the receptors may reflect the immune system’s response to this inhibitory signal. Notably, the MIF-CXCR4 axis has been validated as a central pathway mediating the interaction between macrophages and tumor cells in gastrointestinal stromal tumors, with the expression levels of both MIF and CXCR4 progressively increasing as risk levels rise [79]. In contrast, the present study demonstrates that MIF primarily exerts an immunosuppressive effect in CRC, suggesting that this pathway may fulfill distinct functions under varying TME conditions [80].

In the spatial dimension, the MIF-(CD74+CD44) composite signal exhibits the highest overall communication activity within the spatial communication network and is highly expressed in the tumor core region, thereby confirming the pivotal role of the MIF pathway in promoting tumor invasion [81]. Notably, MIF and CD74 are increasingly being recognized as emerging biomarkers in immune checkpoint blockade (ICB) therapy and are widely considered promising therapeutic targets [55,82,83]. Previous studies have demonstrated that the MIF-CD74 axis promotes CRC progression by modulating cellular metabolism, and that inhibiting this pathway can induce tumor cell death [84]. Furthermore, in both primary and metastatic CRC lesions, MIF-CD74 signaling is dominant in the tumor stem cell population, which may affect tumor progression and metastasis [85,86], which is highly consistent with the conclusions of this study.

Spatial expression mapping further revealed the spatially heterogeneous distribution characteristics of MIF signaling. The spatial complementary distribution of MIF and CD74 suggests that MIF signaling may be directionally transmitted from the tumor core to the immune cell enrichment area via paracrine signaling, thus constructing a directional communication pattern from tumor epithelium to immune cells in a spatial dimension. While the MIF-(CD74+CXCR4) complex signal contributed significantly in single-cell analysis, its activity was relatively low in spatial in situ analysis, suggesting a significant spatial isolation between immune cells expressing CXCR4 and the tumor core region with high MIF expression. This is consistent with the spatial characteristics of the immune rejection phenotype in solid tumors [87]. This discrepancy between single-cell and spatial analysis results highlights the limitations of inferring cell communication solely based on scRNA-seq. The theoretical communication strength calculated based on the average expression level of ligands and receptors may overestimate the actual communication activity constrained by spatial structure [59,88,89].

Together, these single-cell and spatial transcriptomic findings suggest that MIF-centered communication may represent an important regulatory pattern within the CRC TME and may be associated with immune infiltration and immunosuppressive microenvironmental features.

Furthermore, drug sensitivity prediction analysis further revealed a potential association between the expression levels of key genes in the MIF signaling pathway and sensitivity to anti-tumor drugs. Drugs such as temozolomide, MDM2 antagonists, nelarabine, fludarabine, and PI3K inhibitors exhibited lower IC50 values in groups with high expression of *CD74*, *CD44*, or *CXCR4*, suggesting that the expression levels of these receptor molecules may be associated with sensitivity to various targeted therapies. Existing studies have demonstrated that blocking the MDM2-p53 interaction can inhibit tumor formation [90], thereby indirectly supporting our prediction results. Notably, the predicted IC50 values for 5-fluorouracil and Oxaliplatin were significantly lower in patients with high MIF expression compared to the low-expression group; these agents are classic neoadjuvant chemotherapy drugs [91]. To a certain extent, this corroborates the plausibility of a positive correlation between high MIF expression and sensitivity to the aforementioned chemotherapeutic agents, suggesting that MIF expression levels may hold potential clinical value as a predictive biomarker for the efficacy of combination chemotherapy in CRC. However, the aforementioned results are based on exploratory predictions derived from computational models and require further validation through both in vitro and in vivo experiments.

Although the single-cell and spatial multi-omics integration strategy employed in this study enables the analysis of the microenvironment at single-cell resolution and the validation of spatial relationships in situ within tissues, representing the current cutting-edge paradigm in TME research [92,93], certain limitations remain. First, constrained by the completeness of the CRC single-cell reference database, the annotation of certain cell subclusters is difficult to precisely define. Second, current spatial transcriptomics technologies still face limitations regarding whole-transcriptome coverage, rendering them unable to identify all specifically expressed genes within a particular spatial region [94]. Third, this study lacks matched normal colorectal tissue as a control, thereby presenting certain limitations in fully distinguishing between tumor-specific alterations and inherent tissue characteristics. Fourth, although we incorporated existing clinical and molecular annotations for these five scRNA-seq samples, the small cohort size precluded formal stratified statistical analyses based on stage or genotype. Accordingly, the MIF-related signaling patterns identified in this study should be interpreted as hypothesis-generating evidence for candidate therapeutic targets that require validation in larger, clinically annotated CRC cohorts. Moreover, as the *SMAD4* mutation status was not reported in the available raw mutation data tables, it could not be included in the sample-level mutation analysis. Future research should improve cell-subcluster annotation by incorporating more comprehensive CRC single-cell reference datasets and additional experimental or histological validation, apply higher-resolution spatial transcriptomic approaches with broader transcriptome coverage, include matched normal colorectal tissues as controls, and utilize larger cohorts combined with matched histopathological annotations and complete driver gene mutation maps to determine whether MIF signaling activity changes systematically with tumor stage, MS status, mutation status of key CRC driver genes, and other molecular subtypes of CRC. Fifth, this study lacks in vitro and in vivo experimental verification of the function and mechanism of the MIF signaling pathway in CRC. Future studies will include functional validation of the MIF-(CD74+CD44) and MIF-(CD74+CXCR4) signaling axes in CRC cell lines and patient-derived organoids through gene knockdown or pharmacological inhibition, followed by co-culture assays with immune cells to investigate their effects on immune regulation. In vivo validation using xenograft or genetically engineered mouse models will further clarify the therapeutic potential of targeting the MIF pathway in CRC.

## 5. Conclusions

**Figure 9:**
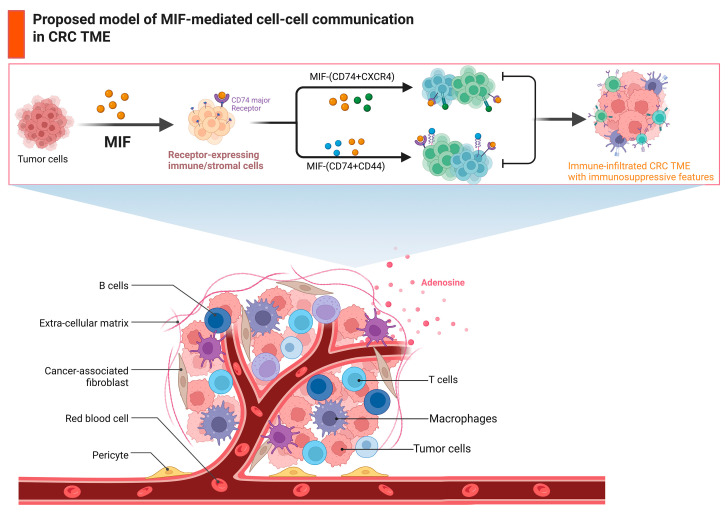
Schematic illustration of the MIF signaling pathway and the proposed working model in the CRC TME. Tumor epithelial cells are proposed to act as an important source of MIF signals. MIF may mediate cell–cell communication through CD74-containing receptor complexes, including MIF-(CD74+CXCR4) and MIF-(CD74+CD44), on receptor-expressing immune and stromal cell populations. Based on the findings of this study, this MIF-centered signaling axis may contribute to an immune-infiltrated CRC tumor microenvironment with immunosuppressive features.

In summary, through an integrated analysis utilizing scRNA-seq and ST technologies, this study systematically elucidated the cellular composition, functional heterogeneity, and spatial distribution characteristics of the TME in primary CRC and identified the pivotal role of the MIF signaling pathway in immune regulation, especially the MIF-(CD74+CD44) and MIF-(CD74+CXCR4) signaling axes. Based on these findings, we proposed a working model in which tumor epithelial cell-derived MIF may mediate cell–cell communication through CD74-containing receptor complexes on receptor-expressing immune and stromal cells, thereby contributing to an immune-infiltrated CRC tumor microenvironment with immunosuppressive features (Figure 9). These findings not only provide novel insights into the pathogenesis of CRC, but also serve as an important reference for future efforts to screen for potential therapeutic targets and develop individualized precision treatment strategies.

## Data Availability

We have shared the link to our data in the manuscript. We used the gene expression data and clinical information data from the TCGA database (http://portal.gdc.cancer.gov/, accessed on 22 May 2024) and the SangerBox platform (http://vip.sangerbox.com/home.html, accessed on 22 May 2024) and the single-cell transcriptomic data, spatial transcriptomic data, and microarray gene expression data from the GEO database (https://www.ncbi.nlm.nih.gov/geo/, accessed on 22 May 2024). Additionally, reference databases utilized for cell annotation, pathway enrichment, intercellular communication, drug sensitivity evaluation, and gene symbol standardization were obtained from CellMarker 2.0 (http://117.50.127.228/CellMarker/, accessed on 14 September 2024), Cell Taxonomy (https://ngdc.cncb.ac.cn/celltaxonomy/, accessed on 14 September 2024), MSigDB (http://www.gsea-msigdb.org/gsea/index.jsp, accessed on 5 March 2025), CellChatDB (https://github.com/sqjin/CellChat, accessed on 20 December 2024), GDSC (https://www.cancerrxgene.org/, accessed on 6 October 2025), and org.Hs.eg.db (https://bioconductor.org/packages/org.Hs.eg.db/, accessed on 3 June 2025). The names of the repository/repositories and accession number(s) can be found within the article. These data are publicly available.

## Supplementary Materials

The following supporting information can be downloaded at: https://www.mdpi.com/article/10.3390/genes17070817/s1, Figure S1. The outgoing signals of each cell type. The line width reflects the strength of the signal. Figure S2. Validation of MIF signaling in GSE241101. (a) Association between the expression of five MIF pathway genes and tumor microenvironment scores in the validation set (* means p<0.05, ** means p<0.01, *** means p<0.001). (b) Spearman correlations of CD74 and CXCR4 expression levels with ImmuneScore and StromalScore in the validation set. Table S1. The human CRC clinical information. Table S2. The marker genes of the subclusters of fibroblast. Table S3. The marker genes of the subclusters of epithelial cells. Table S4. The marker genes of the subclusters of T cells. Table S5. Ranking of ligand–receptor pairs based on total communication strength.

## Abbreviations

The following abbreviations are used in this manuscript:

CRC	Colorectal Cancer
MS	Microsatellite
MSS	microsatellite stable
MSI	microsatellite instability
TME	Tumor Microenvironment
MIF	Macrophage migration inhibitory factor
scRNA-seq	Single-cell RNA sequencing
ST	Spatial Transcriptomics
UMI	unique molecular identifier
UMIs	unique molecular identifiers
CAFs	cancer-associated fibroblasts
GSVA	Gene Set Variation Analysis
GDSC	genomics of drug sensitivity in cancer
IC50	half-maximal inhibitory concentration
HR	hazard ratio
CI	confidence interval
PCA	principal component analysis
UMAP	Uniform Manifold Approximation and Projection
GEO	Gene Expression Omnibus
EMT	epithelial–mesenchymal transition
OS	overall survival
TCGA	The Cancer Genome Atlas
COAD	Colon Adenocarcinoma
CR	normal colorectal tissue
CAC	colon adenocarcinoma
CMAC	colon mucinous adenocarcinoma
