# Supplementary material for: Integrated Single-Cell and Spatial Transcriptomic Analysis Reveals the Immunoregulatory Role of MIF Signaling in Colorectal Cancer
Source: Genes (Basel). 2026 Jul 17;17(7):817. doi: 10.3390/genes17070817 (PMC13409827; doi:10.3390/genes17070817)
Supplement: Supplementary file 1 [file genes-17-00817-s001.zip › genes-4385696-supplementary.pdf]

Supplementary Information for

Integrated Single-Cell and Spatial Transcriptomic Analysis Reveals the Immunoregulatory Role of MIF Signaling in Colorectal Cancer

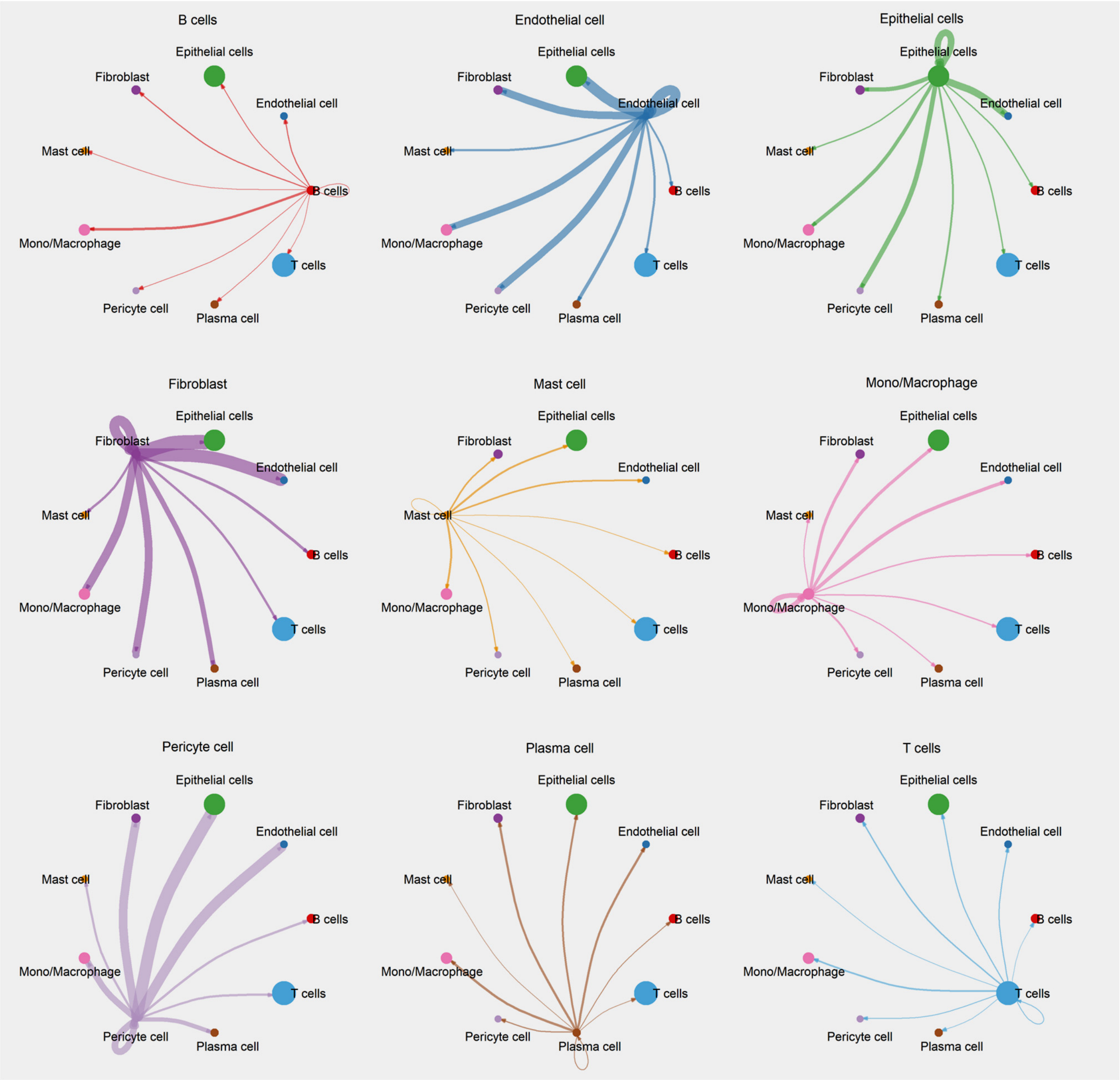

Figure S1. The outgoing signals of each cell type. The line width reflects the strength of the signal.

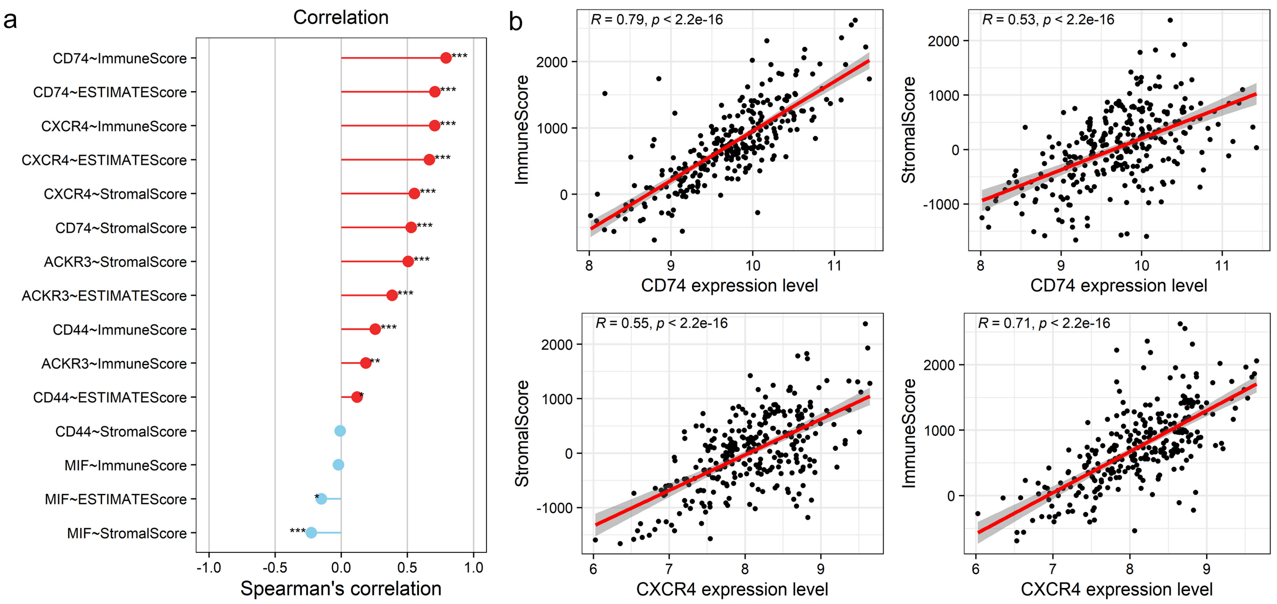

Figure S2. Validation of MIF signaling in GSE241101. (a) Association between the expression of five MIF pathway genes and tumor microenvironment scores in the validation set (* means $P < 0.05$, ** means $P < 0.01$, *** means $P < 0.001$). (b) Spearman correlations of CD74 and CXCR4 expression levels with ImmuneScore and StromalScore in the validation set.

Table S1. The human CRC clinical information.

Patient	C1	C2	C3	C4	C5
GSM accession	GSM7290763	GSM7290769	GSM7290772	GSM7290773	GSM7290774
Sample type	Colorectal cancer	Colorectal cancer	Colorectal cancer	Colorectal cancer	Colorectal cancer
Location	Colon	Colon	Colon	Colon	Colon
Gender	M	M	F	M	M
Current age	47	39	65	85	72
Stage at collection	IV	IV	IV	II	IV
MS status	MSS	MSS	MSS	MSI	MSS
APC	Wildtype	Wildtype	Wildtype	NA	Wildtype
ARID1A	Wildtype	Wildtype	Mutant	NA	Wildtype
ATR	Wildtype	Mutant	Wildtype	NA	Wildtype
AXL	Wildtype	Wildtype	Wildtype	NA	Wildtype
BRAF	Wildtype	Wildtype	Wildtype	NA	Wildtype
FBXW7	Wildtype	Wildtype	Wildtype	NA	Wildtype
KDR	Wildtype	Wildtype	Wildtype	NA	Wildtype
KRAS	Wildtype	Wildtype	Mutant	NA	Mutant
NOTCH2	Mutant	Wildtype	Wildtype	NA	Wildtype
PIK3CA	Wildtype	Wildtype	Wildtype	NA	Wildtype
PPP2R1A	Wildtype	Wildtype	Mutant	NA	Wildtype
PTEN	Wildtype	Wildtype	Wildtype	NA	Wildtype
RNF43	Wildtype	Wildtype	Wildtype	NA	Wildtype
SETD2	Wildtype	Wildtype	Wildtype	NA	Wildtype
TERT	Wildtype	Wildtype	Wildtype	NA	Wildtype
TP53	Mutant	Mutant	Mutant	NA	Wildtype

Table S2. The marker genes of the subclusters of fibroblast.

Cluster	*P* Value	Log2 (Fold change)	Makergene
C0	3.20×10^{-45}	6.93	*IGHA1*
C0	1.83×10^{-36}	5.81	*PPP1R14A*
C0	3.15×10^{-33}	5.45	*EDIL3*
C0	4.89×10^{-30}	5.31	*PTCH1*
C0	2.27×10^{-28}	7.03	*ALKAL2*
C0	5.80×10^{-28}	7.50	*IGLC2*
C0	5.56×10^{-21}	6.32	*EFCC1*
C0	2.92×10^{-19}	6.24	*FRZB*
C0	8.70×10^{-17}	5.95	*TSPAN33*
C0	1.00×10^{-15}	5.24	*ADGRL3*
C0	1.25×10^{-15}	8.39	*ADAMDEC1*
C0	6.28×10^{-15}	6.34	*TRPA1*
C0	2.99×10^{-14}	5.88	*PREX2*
C0	6.80×10^{-13}	6.79	*AGT*
C0	2.24×10^{-12}	5.94	*ABCB1*
C0	9.67×10^{-11}	5.34	*PRR16*
C0	1.58×10^{-09}	5.85	*P2RY14*
C0	2.67×10^{-09}	5.24	*FGFR4*
C0	6.78×10^{-09}	5.25	*F2RL2*
C0	1.15×10^{-08}	5.29	*CXCR4*
C0	1.16×10^{-08}	5.91	*PARM1*
C0	1.28×10^{-07}	6.10	*ADRA2C*
C1	4.32×10^{-96}	7.57	*LIF*
C1	1.17×10^{-75}	5.67	*HAS1*
C1	1.74×10^{-71}	8.11	*HP*
C1	1.86×10^{-60}	6.64	*SLPI*
C1	2.98×10^{-56}	6.82	*AADAC*
C1	7.35×10^{-56}	5.71	*F5*
C1	2.46×10^{-52}	6.07	*BDKRB1*
C1	1.69×10^{-51}	6.64	*PRG4*
C1	7.24×10^{-50}	6.32	*IL6*
C1	3.64×10^{-49}	6.46	*UPK3B*
C1	3.15×10^{-43}	7.47	*KLK11*
C1	1.26×10^{-40}	6.39	*SERPINB2*
C1	1.08×10^{-36}	5.70	*ARC*
C1	1.12×10^{-33}	6.97	*APOA1*

Table S3. The marker genes of the subclusters of epithelial cells.

Cluster	*P* Value	Log2 (Fold change)	Makergene
C0	0	3.68	*PLCG2*
C0	1.73×10^{-12}	3.89	*ANGPTL4*
C0	8.22×10^{-12}	4.68	*LINC01623*
C0	4.81×10^{-11}	3.94	*ZG16*
C0	3.00×10^{-08}	4.02	*CACNG8*
C0	2.77×10^{-06}	3.92	*DMRTA2*
C0	6.10×10^{-06}	4.51	*LINC00243*
C0	6.22×10^{-05}	4.68	*LINC02432*
C0	1.25×10^{-04}	4.04	*TBX2*
C0	2.12×10^{-04}	4.34	*AC010422.1*
C0	2.47×10^{-04}	4.04	*HIST1H2BL*
C0	3.05×10^{-04}	4.18	*TUBB3*
C0	1.35×10^{-03}	4.57	*LINC00923*
C0	1.53×10^{-03}	3.86	*AC004264.1*
C0	4.10×10^{-03}	3.89	*NPIPB11*
C0	4.74×10^{-03}	6.35	*SPRR1B*
C0	6.10×10^{-03}	3.97	*TBX20*
C0	9.26×10^{-03}	4.40	*HIST1H2AD*
C0	6.76×10^{-06}	4.52	*AC097381.1*
C0	9.38×10^{-06}	3.96	*HIST1H2BM*
C1	0	3.89	*EREG*
C1	8.71×10^{-209}	3.86	*F5*
C1	7.78×10^{-190}	4.11	*PLA2G12B*
C1	9.60×10^{-146}	5.25	*SLC13A3*
C1	4.12×10^{-109}	4.25	*HS6ST2*
C1	1.27×10^{-92}	3.85	*PRSS56*
C1	4.25×10^{-79}	3.85	*KRTAP3-1*
C1	1.03×10^{-77}	4.32	*ADAMTSL2*
C1	3.85×10^{-76}	3.93	*STRA6*
C1	6.74×10^{-63}	3.87	*OLFML3*
C1	1.45×10^{-57}	5.00	*TSPAN11*
C1	3.35×10^{-42}	4.65	*WIF1*
C1	1.43×10^{-38}	4.71	*MMP3*
C2	7.72×10^{-54}	2.08	*STMN1*
C2	2.11×10^{-18}	1.81	*GLO1*
C2	2.80×10^{-05}	1.85	*ALG1L*
C2	4.15×10^{-05}	2.67	*NPL*
C2	2.50×10^{-04}	1.82	*LINC00997*
C2	3.43×10^{-04}	2.06	*PIPOX*
C2	5.46×10^{-04}	1.94	*STMND1*
C2	8.31×10^{-04}	1.86	*KIF26A*
C2	8.78×10^{-04}	1.82	*RAB3IL1*

C2	9.58×10^{-04}	1.79	*AL121899.1*
C2	1.03×10^{-03}	1.79	*TDGF1*
C2	1.33×10^{-03}	2.00	*NHSL2*
C3	6.49×10^{-212}	2.92	*KIAA1324*
C3	4.50×10^{-44}	4.61	*ROBO2*
C3	1.11×10^{-34}	5.00	*LSAMP-AS1*
C3	2.77×10^{-26}	4.27	*F11-AS1*
C3	4.49×10^{-20}	4.11	*LINC01918*
C3	1.32×10^{-18}	4.38	*AP001626.1*
C3	2.98×10^{-18}	3.96	*LINC02475*
C3	7.72×10^{-14}	4.92	*BOLL*
C3	7.95×10^{-14}	4.81	*HAPLN1*
C3	7.95×10^{-14}	4.19	*LINC01633*
C3	7.29×10^{-13}	4.68	*AC124066.1*
C3	3.94×10^{-12}	4.38	*AC012615.4*
C3	7.22×10^{-12}	4.49	*COL10A1*
C3	2.28×10^{-10}	3.91	*PCDHA5*
C3	8.85×10^{-10}	4.50	*AC105020.1*
C3	8.85×10^{-10}	4.34	*PCDHB3*
C3	9.04×10^{-10}	4.32	*KCTD8*
C4	0	7.55	*PTPRC*
C4	0	7.25	*TRAC*
C4	0	7.58	*CD2*
C4	3.02×10^{-306}	6.28	*CYTIP*
C4	6.93×10^{-299}	6.89	*TRBC2*
C4	2.52×10^{-287}	8.06	*CCR7*
C4	3.70×10^{-278}	5.64	*CD37*
C4	1.23×10^{-275}	7.22	*SLC2A3*
C4	1.69×10^{-273}	6.25	*LTB*
C4	1.43×10^{-271}	7.08	*GYPC*
C4	6.42×10^{-269}	6.50	*CXCR4*
C4	1.19×10^{-257}	6.91	*SRGN*
C5	2.09×10^{-274}	8.51	*CA4*
C5	3.11×10^{-270}	7.58	*MS4A12*
C5	3.73×10^{-268}	10.83	*GUCA2B*
C5	5.58×10^{-224}	7.55	*SLC26A3*
C5	6.49×10^{-200}	7.11	*CA1*
C5	1.25×10^{-196}	7.01	*CDKN2B-AS1*
C5	3.68×10^{-196}	6.81	*C11orf86*
C5	1.64×10^{-175}	7.54	*TMIGD1*
C5	1.16×10^{-144}	6.02	*EDN3*
C5	5.73×10^{-138}	6.54	*TMEM82*
C5	3.66×10^{-137}	8.82	*OTOP2*
C5	4.40×10^{-128}	5.25	*SCNN1B*

C5	6.04×10^{-125}	5.38	*CD177*
C5	3.41×10^{-124}	6.20	*LINC02023*
C6	0	10.98	*SH2D6*
C6	0	9.86	*LRMP*
C6	0	10.79	*BMX*
C6	0	10.01	*HCK*
C6	0	11.68	*PTGS1*
C6	0	11.10	*HTR3E*
C6	0	12.73	*SH2D7*
C6	0	10.64	*HPGDS*
C6	1.18×10^{-290}	11.18	*GNG13*
C6	3.16×10^{-281}	12.52	*HTR3C*
C6	2.84×10^{-266}	9.96	*PIK3CG*
C6	1.54×10^{-204}	10.39	*MATK*

Table S4. The marker genes of the subclusters of T cells.

Cluster	*P* Value	Log*2* (Fold change)	Makergene
C0	1.73×10^{-142}	3.76	*ICA1*
C0	3.86×10^{-101}	4.48	*FOXP3*
C0	5.35×10^{-66}	3.59	*RTKN2*
C0	7.08×10^{-47}	4.09	*HSPA12A*
C0	1.53×10^{-43}	3.98	*IL1R2*
C0	8.94×10^{-43}	5.56	*FANK1*
C0	6.22×10^{-38}	3.87	*CPM*
C0	1.69×10^{-37}	4.91	*AC017002.3*
C0	4.89×10^{-31}	7.25	*LINC02099*
C0	8.33×10^{-23}	6.03	*CCR8*
C1	1.02×10^{-208}	3.80	*FHIT*
C1	1.84×10^{-140}	3.07	*BACH2*
C1	2.08×10^{-121}	5.10	*AK5*
C1	5.49×10^{-94}	3.39	*LYPD3*
C1	2.02×10^{-42}	6.32	*MMP28*
C1	1.36×10^{-29}	3.50	*GTSCR1*
C2	0	7.18	*KLRD1*
C2	3.22×10^{-278}	6.73	*GZMH*
C2	2.51×10^{-198}	6.93	*GNLY*
C2	8.58×10^{-196}	7.32	*XCL2*
C2	3.87×10^{-159}	5.99	*CCL3*
C2	6.11×10^{-144}	5.95	*CCL3L1*
C2	4.29×10^{-131}	7.83	*KLRC2*
C2	4.08×10^{-107}	8.29	*KIR2DL4*
C2	1.70×10^{-99}	5.94	*KLRC1*
C2	6.02×10^{-93}	6.90	*FCGR3A*
C2	1.94×10^{-87}	6.01	*KLRC4*
C2	1.07×10^{-85}	8.27	*VCAM1*
C2	2.85×10^{-80}	7.36	*KLRF1*
C2	6.02×10^{-79}	6.33	*AC243829.4*
C2	1.81×10^{-67}	6.30	*ATP8B4*
C2	3.62×10^{-57}	6.37	*NCR1*
C2	2.73×10^{-56}	8.16	*FGFBP2*
C2	7.16×10^{-48}	6.15	*FCRL6*
C3	1.55×10^{-51}	3.33	*IL4I1*
C3	5.52×10^{-44}	5.48	*LINC01644*
C3	3.26×10^{-43}	5.65	*LTK*
C3	1.05×10^{-38}	3.55	*RORC*
C3	5.30×10^{-34}	3.26	*CYB561*
C3	2.25×10^{-32}	4.64	*SLC4A10*
C3	4.79×10^{-31}	3.36	*B3GALT2*

Table S5. Ranking of ligand-receptor pairs based on total communication strength.

Ligand-ReceptorPair	Total Communication Strength	Rank
MIF-(CD74+CD44)	26091.56	1
APP-CD74	19612.77	2
COL1A2-(ITGA3+ITGB1)	14852.58	3
CEACAM1-CEACAM5	14654.00	4
CDH1-CDH1	14357.19	5
COL1A2-(ITGA2+ITGB1)	13433.81	6
COL1A2-CD44	12981.76	7
CDH1-(ITGA2+ITGB1)	12701.59	8
COL1A2-SDC1	11543.11	9
LAMB1-(ITGA6+ITGB1)	11393.10	10
CD99-CD99	10011.60	11
COL1A1-(ITGA3+ITGB1)	9175.55	12
COL1A2-SDC4	9136.55	13
COL1A2-(ITGA9+ITGB1)	8699.62	14
LAMB1-(ITGA6+ITGB4)	8538.36	15
COL1A1-(ITGA2+ITGB1)	8376.15	16
LAMC1-(ITGA6+ITGB1)	8195.47	17
LAMB1-(ITGA3+ITGB1)	8103.51	18
COL1A1-CD44	7996.29	19
MDK-(ITGA6+ITGB1)	7735.60	20
LAMB3-(ITGA6+ITGB1)	7722.51	21
FN1-(ITGA3+ITGB1)	7626.16	22
LAMA4-(ITGA6+ITGB1)	7563.30	23
LAMC2-(ITGA6+ITGB1)	7515.60	24
LAMB1-(ITGA2+ITGB1)	7356.58	25
COL1A2-(ITGA1+ITGB1)	7289.89	26
MIF-(CD74+CXCR4)	7091.62	27
COL1A1-SDC1	7072.06	28
LAMB1-CD44	7030.49	29
COL6A3-(ITGA3+ITGB1)	6784.61	30
FN1-CD44	6758.85	31
LAMA3-(ITGA6+ITGB1)	6744.77	32
MDK-NCL	6649.47	33
COL6A3-(ITGA2+ITGB1)	6160.78	34
LAMC1-(ITGA6+ITGB4)	6121.00	35

COL6A2-(ITGA3+ITGB1)	6003.84	36
FN1-SDC1	5981.21	37
LAMC1-(ITGA3+ITGB1)	5908.67	38
COL6A3-CD44	5891.40	39
LAMB3-(ITGA6+ITGB4)	5856.85	40
LAMB2-(ITGA6+ITGB1)	5838.41	41
LAMA5-(ITGA6+ITGB1)	5756.10	42
LAMC2-(ITGA6+ITGB4)	5727.89	43
COL1A1-SDC4	5645.45	44
LAMA4-(ITGA6+ITGB4)	5622.02	45
LAMB3-(ITGA3+ITGB1)	5609.23	46
HLA-C-CD8A	5584.58	47
LAMA2-(ITGA6+ITGB1)	5546.82	48
COL4A1-(ITGA3+ITGB1)	5493.27	49
LAMA4-(ITGA3+ITGB1)	5470.79	50
LAMC2-(ITGA3+ITGB1)	5454.02	51
THBS1-(ITGA3+ITGB1)	5446.89	52
COL6A2-(ITGA2+ITGB1)	5397.09	53
COL1A1-(ITGA9+ITGB1)	5370.70	54
LAMC1-(ITGA2+ITGB1)	5307.02	55
COL6A3-SDC1	5227.65	56
COL6A2-CD44	5160.03	57
LAMA3-(ITGA6+ITGB4)	5143.82	58
LAMC1-CD44	5064.11	59
FN1-(ITGAV+ITGB1)	5046.47	60
LAMB3-(ITGA2+ITGB1)	5042.13	61
COL4A1-(ITGA2+ITGB1)	5006.00	62
COL9A2-(ITGA3+ITGB1)	4985.80	63
COL6A1-(ITGA3+ITGB1)	4960.35	64
LAMC2-(ITGA2+ITGB1)	4935.58	65
LAMA3-(ITGA3+ITGB1)	4893.43	66
LAMA4-(ITGA2+ITGB1)	4880.24	67
COL4A1-CD44	4751.04	68
LAMB3-CD44	4750.96	69
LAMB1-(ITGA9+ITGB1)	4746.79	70
FN1-SDC4	4734.88	71
LAMA4-CD44	4680.32	72

LAMC2-CD44	4627.89	73
COL6A2-SDC1	4612.45	74
COL1A1-(ITGA1+ITGB1)	4610.34	75
COL6A1-(ITGA2+ITGB1)	4490.67	76
CD99-CD99L2	4479.30	77
LAMA3-(ITGA2+ITGB1)	4432.59	78
CD8A-CEACAM5	4401.47	79
COL9A2-(ITGA2+ITGB1)	4341.87	80
LAMB2-(ITGA6+ITGB4)	4330.62	81
HLA-A-CD8A	4318.10	82
LAMA5-(ITGA6+ITGB4)	4303.02	83
COL6A1-CD44	4268.34	84
NAMPT-INSR	4261.39	85
MDK-SDC1	4220.95	86
COL9A2-CD44	4220.23	87
LAMB2-(ITGA3+ITGB1)	4213.41	88
COL4A1-SDC1	4198.27	89
THBS1-SDC1	4164.26	90
LAMA5-(ITGA3+ITGB1)	4162.55	91
COL6A3-SDC4	4160.29	92
LAMA3-CD44	4148.76	93
LAMA2-(ITGA6+ITGB4)	4097.01	94
SEMA4D-PLXNB2	4089.39	95
LAMA2-(ITGA3+ITGB1)	3998.12	96
COL6A3-(ITGA9+ITGB1)	3968.54	97
LAMB1-(ITGA1+ITGB1)	3935.21	98
COL9A2-SDC1	3857.98	99
COL6A1-SDC1	3794.61	100
COL4A2-(ITGA3+ITGB1)	3793.14	101
LAMB2-(ITGA2+ITGB1)	3770.98	102
LAMA5-(ITGA2+ITGB1)	3743.45	103
SEMA4C-PLXNB2	3721.09	104
COL6A2-SDC4	3653.26	105
LAMB2-CD44	3620.85	106
COL1A2-(ITGAV+ITGB8)	3598.01	107
LAMA2-(ITGA2+ITGB1)	3558.34	108
LAMA5-CD44	3554.69	109

COL6A2-(ITGA9+ITGB1)	3462.89	110
COL6A3-(ITGA1+ITGB1)	3446.57	111
COL4A2-(ITGA2+ITGB1)	3445.89	112
LAMA2-CD44	3438.27	113
LAMC1-(ITGA9+ITGB1)	3419.50	114
MDK-SDC4	3380.21	115
CDH1-(ITGAE+ITGB7)	3367.55	116
COL4A1-SDC4	3360.41	117
SEMA4G-PLXNB2	3341.23	118
THBS1-SDC4	3310.50	119
HLA-E-CD8A	3283.00	120
ADGRE5-CD55	3274.39	121
COL4A2-CD44	3271.97	122
EFNA2-EPHA2	3239.20	123
COL4A5-(ITGA3+ITGB1)	3232.38	124
COL4A1-(ITGA9+ITGB1)	3203.50	125
LAMA4-(ITGA9+ITGB1)	3165.11	126
LAMB3-(ITGA9+ITGB1)	3113.13	127
COL6A2-(ITGA1+ITGB1)	3062.86	128
LAMB1-DAG1	3039.05	129
COL6A1-SDC4	3021.99	130
LAMC2-(ITGA9+ITGB1)	3000.00	131
PRSS3-PARD3	2995.01	132
MDK-LRP1	2993.89	133
COL9A2-SDC4	2989.84	134
NAMPT-(ITGA5+ITGB1)	2947.58	135
LAMC1-(ITGA1+ITGB1)	2931.06	136
COL4A2-SDC1	2898.54	137
COL6A1-(ITGA9+ITGB1)	2871.07	138
COL4A5-(ITGA2+ITGB1)	2861.31	139
EFNB1-EPHB4	2846.27	140
COL4A1-(ITGA1+ITGB1)	2833.62	141
JAM2-(ITGA3+ITGB1)	2832.33	142
AGRN-DAG1	2829.32	143
EFNB1-EPHB2	2828.35	144
LAMA4-(ITGA1+ITGB1)	2765.40	145
COL9A2-(ITGA9+ITGB1)	2749.75	146

EFNA1-EPHA2	2744.15	147
COL4A5-CD44	2742.86	148
SEMA4A-PLXNB2	2717.93	149
HLA-F-CD8A	2707.73	150
LAMA3-(ITGA9+ITGB1)	2702.61	151
FN1-(ITGA4+ITGB1)	2679.06	152
LAMB3-(ITGA1+ITGB1)	2606.93	153
GRN-SORT1	2579.78	154
COL6A1-(ITGA1+ITGB1)	2564.93	155
LAMC2-(ITGA1+ITGB1)	2534.17	156
CD46-JAG1	2519.30	157
COL4A5-SDC1	2469.47	158
EFNB2-EPHB4	2468.73	159
EFNB2-EPHB2	2458.97	160
LAMB2-(ITGA9+ITGB1)	2451.92	161
OCLN-OCLN	2425.36	162
CD209-CEACAM1	2406.48	163
CD1D-CEACAM5	2403.53	164
LAMA5-(ITGA9+ITGB1)	2401.46	165
LAMA2-(ITGA9+ITGB1)	2360.85	166
COL4A2-SDC4	2313.56	167
FN1-(ITGA5+ITGB1)	2311.36	168
COL9A2-(ITGA1+ITGB1)	2287.41	169
LAMA3-(ITGA1+ITGB1)	2285.41	170
PRSS3-F2R	2284.32	171
F11R-F11R	2268.29	172
HSPG2-DAG1	2260.66	173
COL1A1-(ITGAV+ITGB8)	2256.67	174
IGF1-(ITGA6+ITGB4)	2234.98	175
COL4A2-(ITGA9+ITGB1)	2207.53	176
TNFSF10-TNFRSF10A	2188.81	177
LAMC1-DAG1	2175.85	178
LAMB2-(ITGA1+ITGB1)	2168.69	179
COL1A2-(ITGA11+ITGB1)	2166.74	180
FN1-(ITGAV+ITGB6)	2111.98	181
LAMA5-(ITGA1+ITGB1)	2098.82	182
AREG-(EGFR+ERBB2)	2078.55	183

THBS1-CD47	2076.53	184
LAMB3-DAG1	2052.80	185
DSC2-DSG2	2047.23	186
LAMA2-(ITGA1+ITGB1)	2026.18	187
LAMA4-DAG1	2005.69	188
LAMC2-DAG1	1997.70	189
COL4A2-(ITGA1+ITGB1)	1995.84	190
LAMB1-(ITGAV+ITGB8)	1991.69	191
NECTIN3-PVR	1971.76	192
FN1-(ITGAV+ITGB8)	1953.16	193
COL4A5-SDC4	1938.50	194
MDK-(ITGA4+ITGB1)	1932.94	195
MIF-ACKR3	1922.48	196
PDGFA-PDGFRB	1902.08	197
TNXB-SDC1	1886.49	198
JAM2-(ITGAV+ITGB1)	1839.45	199
COL4A5-(ITGA9+ITGB1)	1833.30	200
